# Anthropogenic extinction threats and future loss of evolutionary history in reef corals

**DOI:** 10.1002/ece3.527

**Published:** 2013-03-18

**Authors:** Danwei Huang, Kaustuv Roy

**Affiliations:** 1 Department of Biological Sciences, National University of SingaporeSingapore, 117543; 2 Scripps Institution of Oceanography, University of California San DiegoLa Jolla, California, 92093; 3 Section of Ecology, Behavior and Evolution, University of California San DiegoLa Jolla, California, 92093

**Keywords:** Phylogenetic conservatism, phylogenetic diversity, Scleractinia, supertree, tree shape

## Abstract

Extinction always results in loss of phylogenetic diversity (PD), but phylogenetically selective extinctions have long been thought to disproportionately reduce PD. Recent simulations show that tree shapes also play an important role in determining the magnitude of PD loss, potentially offsetting the effects of clustered extinctions. While patterns of PD loss under different extinction scenarios are becoming well characterized in model phylogenies, analyses of real clades that often have unbalanced tree shapes remain scarce, particularly for marine organisms. Here, we use a fossil-calibrated phylogeny of all living scleractinian reef corals in conjunction with IUCN data on extinction vulnerabilities to quantify how loss of species in different threat categories will affect the PD of this group. Our analyses reveal that predicted PD loss in corals varies substantially among different threats, with extinctions due to bleaching and disease having the largest negative effects on PD. In general, more phylogenetically clustered extinctions lead to larger losses of PD in corals, but there are notable exceptions; extinction of rare corals from distantly-related old and unique lineages can also result in substantial PD loss. Thus our results show that loss of PD in reef corals is dependent on both tree shape and the nature of extinction threats.

## Introduction

Extinction inevitably leads to loss of evolutionary diversity. As more species become impacted by anthropogenic activities, understanding the amount of evolutionary history that may be at risk has become an important component of conservation planning (Faith 1992; Purvis et al. 2000; Rodrigues and Gaston 2002; Mooers and Atkins 2003; Rolland et al. 2012). Changes in phylogenetic diversity (PD), defined as the total branch length in a phylogeny (Faith 1992), is the metric widely used for quantifying such loss. When extinction is random, loss of PD can be surprisingly small even when extinction rates are high (Nee and May 1997; see also Raup et al. 1973). In contrast, phylogenetically selective extinction, where closely related species are preferentially removed, generally leads to a disproportionately large loss of PD (McKinney 1997; Russell et al. 1998; Purvis et al. 2000; Corey and Waite 2008; Purvis 2008; Roy et al. 2009). However, the amount of PD lost can also depend on the distribution of branch lengths among the species involved (Mooers et al. 2012), and simulation studies have shown that for certain tree shapes, PD loss is minimized even in the case of nonrandom losses (Heard and Mooers 2000; Parhar and Mooers 2011). While patterns of PD loss for simulated trees under different extinction scenarios are becoming well characterized (Parhar and Mooers 2011), predictions for real-world phylogenies that often have unbalanced shapes (Mooers 1995; Purvis and Agapow 2002; Blum and François 2006; Purvis et al. 2011; Huang 2012) remain scarce.

Empirical analyses using the IUCN Red List of Threatened Species (IUCN 2001; Mace et al. 2008) generally predict substantial losses of future PD due to anthropogenic extinctions (Purvis et al. 2000; von Euler 2001; Mooers and Atkins 2003; Davies et al. 2008). However, these studies have focused primarily on terrestrial vertebrates. Despite the multitude of human impacts on marine biodiversity (Worm et al. 2006; Halpern et al. 2008; Hoegh-Guldberg and Bruno 2010), we know very little about how anthropogenic extinctions are likely to affect the PD of marine clades (see Huang 2012). Moreover, existing estimates of future changes in PD tend to be largely based on aggregate losses of species on various Red List categories rather than extinctions due to specific threats (Purvis et al. 2000; von Euler 2001; Mooers and Atkins 2003). Given that extinction selectivities can vary with extinction agents (Chiba and Roy 2011; Bromham et al. 2012), knowledge of how PD is affected by individual threats should be a useful conservation tool – threats that are likely to result in large losses of PD are arguably more detrimental from an evolutionary standpoint compared with those with more limited impacts on PD.

Reef-building corals not only represent a critical component of marine biodiversity but also provide important ecosystem services to an estimated 450 million people from 109 countries (Pandolfi et al. 2011). This group is being heavily impacted by a multitude of anthropogenic impacts, ranging from ocean acidification to pollution and over-harvesting (Hughes et al. 2003; Bruno et al. 2007; Carpenter et al. 2008), and a substantial number of coral species are vulnerable to threats such as bleaching (loss of algal endosymbiont *Symbiodinium*; 41.9%), disease (31.0%), and outbreak of crown-of-thorns seastar (CoTs; Acanthaster planci; 27.3%) (Carpenter et al. 2008). As a result, many recent models predict dramatic global-scale losses of coral species in the foreseeable future (e.g., Hoegh-Guldberg et al. 2007; Buddemeier et al. 2011; Frieler et al. 2013).

A recent study showed that anthropogenic threats to coral reefs are not evenly distributed across the coral phylogeny – some threats are more clustered on the tree compared with others (Huang 2012). The study also revealed that species in individual Red List categories are not closely related, and extinction of these corals results in smaller than random losses of PD. However, a major limitation of those analyses was the use of an uncalibrated phylogeny, where PD values were based on molecular sequence variations rather than time-calibrated branch lengths. In addition, while Huang (2012) showed that individual threats such as bleaching, disease, and CoTs predation were phylogenetically clustered, the relationship between levels of such clustering and degree of PD loss has never been quantified.

In this study we build on the work of Huang (2012) and use 1000 fossil-calibrated, fully-resolved time trees representing all 838 living reef-building coral species to quantify changes in PD not only due to losses of species with different levels of vulnerabilities but also from different types of threats, as identified by the IUCN. In addition, we use these data to test the hypothesis that phylogenetic clustering of extinctions is the best predictor of the loss of PD due to anthropogenic extinctions (see Parhar and Mooers 2011). Because the reef coral tree is comprised of two major clades that diverged during the Paleozoic, we ran each analysis separately for each clade to explore clade-specific effects. Finally, we investigate the effects of the constraints used to reconstruct the coral supertree on computations of tree shape and loss of PD by carrying out Markov chain Monte Carlo (MCMC) tree estimation based solely on a mtDNA dataset with only the fossil node calibrations.

## Material and Methods

We used the supertree method (Baum 1992; Ragan 1992) detailed in Huang (2012) to reconstruct a phylogeny comprising 975 scleractinian coral terminals, of which 838 were reef species, including 827 species evaluated for the IUCN Red List of Threatened Species (Carpenter et al. 2008). The rest were nonreef corals with a mixture of species- and genus-level terminals (Table S1). We updated the mitochondrial DNA dataset from Huang (2012) to assemble a 466-species matrix that was analyzed via the maximum likelihood criterion using RAxML 7.2.8 (Stamatakis 2006; Stamatakis et al. 2008; Miller et al. 2010), based on the partitioned GTRGAMMA model, 1000 alternate parsimony starting trees, and 1000 bootstrap pseudoreplicates. The molecular phylogeny obtained here, plus morphological trees, and taxonomic sources in Huang (2012) were each coded into bootstrap percentage-weighted matrix representation with parsimony (MRP) using SuperMRP 1.2.1 (Bininda-Emonds et al. 2005). We combined the MRPs from all sources to generate a 757-character dataset, and ran maximum parsimony analysis with 10,000 random additions (rearrangement limit of 10^9^ per replicate) using PAUP* 4.0b10 (Swofford 2003). Ten thousand trees with the shortest length were subjected to further tree-bisection-reconnection to obtain a total of 17,943 minimum length trees that were summarized as a strict consensus cladogram (available in TreeBASE).

We used BEAST 1.6.2 (Drummond and Rambaut 2007) to fit the molecular data onto the cladogram using fossil node calibrations in Simpson et al. (2011) and Stolarski et al. (2011). Maximum bound for root height was extended from 271 million years ago (Mya) (Simpson et al. 2011) to 455 Mya in order to incorporate the earlier origin of Scleractinia estimated by Stolarski et al. (2011). We carried out five MCMC analyses of 30 million generations with a sampling interval of 1000. Runs were combined and we discarded the first one-third of all posterior trees as burn-in after checking for convergence using Tracer 1.5 (Rambaut and Drummond 2009). Polytomies were randomly resolved to generate 1000 trees using PolytomyResolver (Kuhn et al. 2011). We incorporated uncertainty of node age estimates by placing normal prior constraints based on the 95% highest posterior density (HPD) obtained through the data fitting process above (see Collen et al. 2011). Five analyses of 3 million generations were combined and, following rejection of the first one-third of trees, subsampled every 10,000 iterations.

We evaluated tree shape of the supertrees relative to the Yule model using gamma (Pybus and Harvey 2000) and Colless statistics (Colless 1982). A negative gamma value indicates that edges near the tips are generally longer than those close to the root, and the reverse with positive gamma. The Colless' index of each coral supertree, representing the sum of size disparity between the two daughter clades at each node, was compared with 1000 randomly generated Yule trees. A larger Colless' index indicates greater imbalance.

We characterized the clustering of extinction risks with the *D* statistic (Fritz and Purvis 2010a), representing the sum of sister-clade disparities (*D* = 0, clumped; *D* = 1, random). This was computed in R package CAIC 1.0.4 (Orme et al. 2008) separately for each of the eight attributes assessed as part of the IUCN Red List of reef corals (IUCN 2001; Carpenter et al. 2008). Three of these represent the conservation status of species – Endangered (EN) and above, Vulnerable (VU) and above, and Near Threatened (NT) and above. The other five represent traits or impacts that have been identified by the IUCN as known correlates of extinction vulnerability in reef corals – rarity, restricted or highly fragmented range, moderate/high susceptibility to bleaching, disease, and CoTs predation (Carpenter et al. 2008; Mace et al. 2008). For each of these cases, we calculated the projected loss of PD assuming that all species with that trait will go extinct, and compared it with the loss under a null model of random extinction with the same number of species (1000 replicates) (Sechrest 2002; Fritz and Purvis 2010b). We used the metric percentage difference in projected PD [%ΔE(PD)], equation (4) in Parhar and Mooers (2011), to quantify the difference between observed and random PD declines.

As the reef coral phylogeny is comprised of two major clades (termed the complex and robust clades) diverging from a deep root (mean age 365.3 Mya, 95% HPD 308.4–422.8 Mya), it is possible that these two edges may contain an overwhelmingly large proportion of total PD, thereby obscuring the patterns within each clade. To investigate this issue, we repeated all analyses separately for each of the complex and robust clades.

To explore potential effects of the constraints used to construct the supertree on computations of tree shape and loss of PD, we undertook MCMC tree estimation based on the mtDNA dataset with only the fossil node calibrations (i.e., without the constraints from the strict consensus cladogram). We simulated 30 million generations with a sampling interval of 1000. The final one-third of trees obtained were retained and subsampled every 10,000 iterations, resulting in 1000 trees that were analyzed as above.

## Results and Discussion

The 1000 fully-resolved coral supertrees register values of the Colless' index that are significantly higher than predicted by the Yule model (*P* < 0.01), indicating that they are more asymmetric than the Yule expectation. Furthermore, the coral phylogenies have large positive values of the gamma statistic (28.63 ± SD 0.94), greater than the standard normal distribution centered on zero, that is, characteristic of Yule trees (Pybus and Harvey 2000). Note that we have restricted our calculations to reef coral species because, few nonreef corals have been assessed for their conservation status, but the tree shape statistics presented here are likely to be conservative as improvement of taxon sampling generally leads to higher gamma (Pybus and Harvey 2000; Pybus et al. 2002). In fact, the gamma value here already exceeds the −8.09 to 4.96 range recorded in other animals and plants (McPeek 2008; see also Morlon et al. 2010; Simpson et al. 2011). Even the minimally-constrained mtDNA trees, despite sampling only 43.7% of all reef corals, have a mean gamma of 18.15 (± SD 0.65), and are significantly more unbalanced than Yule trees according to the Colless' index (*P* < 0.01). Furthermore, the supertrees trimmed to the same tips as the mtDNA trees gave lower but comparable gamma values (17.30 ± SD 0.58), showing that the extreme imbalance of the coral phylogeny is unlikely to be an artifact of the constrained supertree.

**Figure 1:**
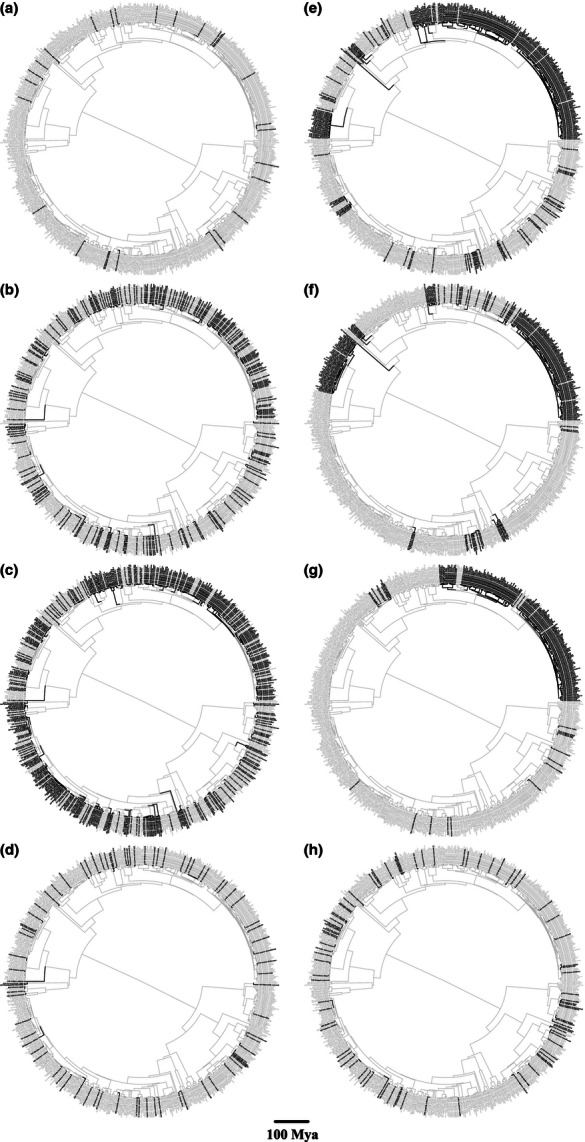
Time-calibrated reef coral phylogeny (first of 1000) with darker branches denoting the phylogenetic distribution of species in each of the following categories: (A) Endangered and above, (B) Vulnerable and above, (C) Near Threatened and above, (D) rare, (E) susceptible to bleaching, (F) susceptible to disease, (G) susceptible to *Acanthaster planci* predation, and (H) with restricted or fragmented range.

**Figure 2:**
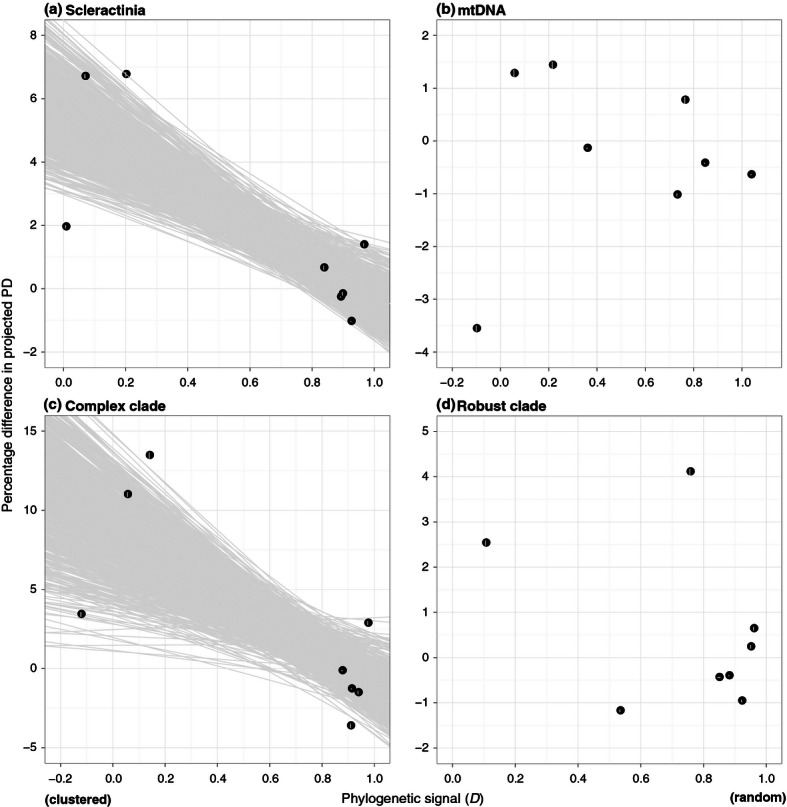
Relationships between projected loss of coral phylogenetic diversity (relative to random extinction) and phylogenetic clustering, that is, lower D (Fritz and Purvis 2010a), for (A) Scleractinia supertrees, (B) mtDNA trees (43.7% of species sampled), (C) complex clade (55.1% of species), and (D) robust clade (44.9% of species). Data points represent mean %*ΔE(PD)* (Parhar and Mooers 2011) for each of the eight extinction scenarios modeled here. Bars within each point represent 95% confidence intervals. Regression lines for the 1000 fully-resolved trees are shown in light gray only for datasets that show significant linear relationship.

**Figure 3:**
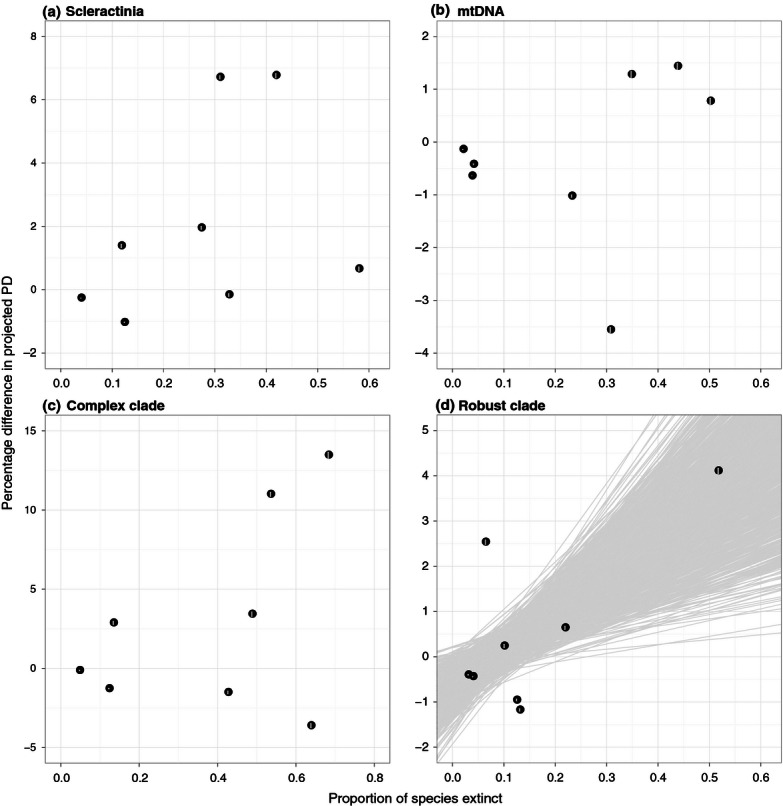
Relationships between projected loss of coral phylogenetic diversity (relative to random extinction) and proportion of species in each extinction category. See Figure 2 for plot details.

Phylogenetic distributions of the eight forms of extinction vulnerability are shown in Figure 1; some of these categories are highly clustered on the tree while others are widely dispersed. Projected changes in PD due to extinction also vary widely among categories (Table 1, Fig. 2A and B). The largest %ΔE(PD) is associated with coral bleaching, with losses due to disease a close second. On the other hand, losing corals with restricted or highly fragmented ranges leads to less PD loss than expected. %ΔE(PD) is not significantly related to the proportion of species in each extinction category (*R*^*2*^ = 0.142; Fig. 3A and B), but overall there is a significant negative correlation between projected excess loss of PD and *D* (*R*^*2*^ = 0.584, slope = −5.70, *P* = 0.038; Fig. 2A). In other words, higher phylogenetic clustering of extinction threats (lower *D*) leads to greater declines of PD relative to random extinctions.

**Table 1:** Traits examined in this study

Threat	Percentage of species	D	Percentage difference in projected PD
Scleractinia (gamma = 28.63 ± SD 0.94)			
Endangered and above	3.92	*0.891 ± 0.035*	−0.263 ± 0.278
Vulnerable and above	32.70	0.897 ± 0.019	−0.162 ± 0.927
Near Threatened and above	57.99	0.837 ± 0.017	0.657 ± 1.177
Rare	11.77	*0.966 ± 0.024*	1.387 ± 0.954
Susceptible to bleaching	41.86	0.200 ± 0.010	6.766 ± 1.254
Susceptible to disease	30.96	**0.069 ± 0.011**	6.706 ± 1.083
Susceptible to CoTs predation	27.33	**0.007 ± 0.011**	1.953 ± 0.987
Restricted/fragmented range	12.35	*0.925 ± 0.026*	−1.033 ± 0.378
mtDNA (gamma = 18.15 ± SD 0.65)			
Endangered and above	2.03	**0.359 ± 0.069**	−0.138 ± 0.136
Vulnerable and above	23.19	0.731 ± 0.025	−1.023 ± 0.527
Near Threatened and above	50.14	0.763 ± 0.019	0.774 ± 0.979
Rare	3.77	*1.038 ± 0.061*	−0.641 ± 0.165
Susceptible to bleaching	43.77	**0.215 ± 0.010**	1.435 ± 1.076
Susceptible to disease	34.78	**0.056 ± 0.012**	1.279 ± 1.091
Susceptible to CoTs predation	30.72	**−0.100 ± 0.011**	−3.557 ± 0.910
Restricted/fragmented range	4.06	*0.845 ± 0.047*	−0.421 ± 0.221
Complex clade (gamma = 19.84 ± SD 0.93)			
Endangered and above	4.74	*0.876 ± 0.059*	−0.135 ± 0.572
Vulnerable and above	42.62	*0.938 ± 0.034*	−1.521 ± 1.965
Near Threatened and above	63.79	*0.909 ± 0.033*	−3.621 ± 2.495
Rare	13.37	*0.975 ± 0.038*	2.868 ± 1.976
Susceptible to bleaching	68.25	**0.139 ± 0.018**	13.461 ± 3.227
Susceptible to disease	53.48	**0.055 ± 0.020**	10.994 ± 2.589
Susceptible to CoTs predation	48.75	**−0.122 ± 0.017**	3.422 ± 2.539
Restricted/fragmented range	12.26	*0.912 ± 0.048*	−1.284 ± 0.678
Robust clade (gamma = 18.31 ± SD 0.69)			
Endangered and above	3.04	*0.881 ± 0.041*	−0.402 ± 0.215
Vulnerable and above	21.88	*0.959 ± 0.026*	0.640 ± 0.748
Near Threatened and above	51.67	0.757 ± 0.022	4.107 ± 1.139
Rare	10.03	*0.950 ± 0.033*	0.238 ± 0.659
Susceptible to bleaching	13.07	0.533 ± 0.030	−1.177 ± 0.502
Susceptible to disease	6.38	**0.105 ± 0.013**	2.534 ± 0.954
Susceptible to CoTs predation	3.95	*0.849 ± 0.087*	−0.439 ± 0.294
Restricted/fragmented range	12.46	*0.921 ± 0.029*	−0.959 ± 0.481

*D* in italics denotes *P* > 0.01: trait not highly significantly different from random (*D* = 1). *D* in bold denotes *P* > 0.01: trait not highly significantly different from clumped (*D* = 0). All projected PD significantly different from random (± SD).

Analyses of individual clades show that in terms of shape, both clades are more unbalanced than the Yule model, with Colless' index being significantly higher than the Yule expectation (*P* < 0.01). Gamma values are also comparable – mean 19.84 ± SD 0.93 for the complex clade and 18.31 ± SD 0.69 for the robust clade. However, while the overall correlation between PD loss and *D* is evident in the complex clade (*R*^*2*^ = 0.519, slope = −9.83, *P* = 0.068; Fig. 2C), it is not significant within the robust clade (*R*^*2*^ = 0.155; Fig. 2D). Interestingly, the proportion of species in each extinction category better explains the variation in PD loss for the latter group (*R*^*2*^ = 0.465, slope = 8.21, *P* = 0.099; Fig. 3C and D).

A weak positive association between phylogenetic clustering of extinction threats and projected loss of PD has been shown for simulated Yule trees (Parhar and Mooers 2011), and this trend holds for corals in general. However, our results show that this relationship is only present in one of the two major clades. For the robust clade, excess loss of PD is positively correlated with level of extinction but not with clustering of threats (Figs. 2D, 3D). This distinct behavior appears unrelated to tree shape, and lends support to the notion that patterns of PD declines can be taxon specific.

Overall, phylogenetic clustering is not a good predictor of extinction categories with extreme *D* values. The most phylogenetically clustered threat (susceptible to CoTs predation) on our trees has a depressed excess loss of PD, while the least clustered trait (rarity) has elevated excess PD loss (Table 1; Fig. 2A). This discrepancy is largely due to positional differences among threats on the tree. For example, the phylogenetic placement of corals vulnerable to CoTs predation is similar to that of bleaching-susceptible species, with *Acropora* and *Montipora* being affected by both risk factors. However, a neighboring clade *Alveopora* and a distant group *Goniopora* are affected by bleaching but not CoTs, strengthening the phylogenetic signal of the latter. This pattern is also present for the mtDNA trees, with an even more extreme low relative value of projected PD loss than expected due to CoTs predation (Fig. 2B). Intensification of bleaching outbreaks could result in additional elimination of the entire *Alveopora* (mean age 60.6 Mya, 95% HPD 43.8–78.6 Mya) and *Goniopora* (mean age 49.1 Mya, 95% HPD 16.9–91.7 Mya) clades, representing substantial PD losses not associated with CoTs predation.

At the other end of the spectrum, rare species and those with restricted or highly fragmented ranges have the least clustered distribution on the coral tree (Table 1; Fig. 1). Yet losing rare corals leads to a much larger loss of PD compared with range-restricted taxa simply because, the former includes species that are relatively old and phylogenetically unique, such as *Poritipora paliformis* (mean age 89.0 ± SD 36.8 Mya) and *Montastraea salebrosa* (monospecific lineage sister to 41 species).

Thus, while our results are generally consistent with the idea that phylogenetic clustering of extinction leads to a higher loss of PD than expected, they also highlight the critical role of tree shape in determining the magnitude of PD loss (Heard and Mooers 2000; Parhar and Mooers 2011). The relationship between phylogenetic signal and projected loss of PD in reef corals is similar to that for the ‘extreme case of diversification’ modeled by Parhar and Mooers (2011). On one hand, phylogenetically clustered extinctions of reef coral species due to bleaching (41.9%), disease (31.0%), and CoTs predation (27.3%) have the potential to obliterate a considerable amount of the evolutionary history of this group, on the other, extinction of rare species that are scattered across the phylogeny can also lead to the loss of many extraordinary lineages that are old and unique, thereby eliminating substantial PD. In contrast, the loss of range-restricted species, generally considered to be of conservation concern, has a relatively small effect on the total PD of reef corals.

To date, the pattern of PD loss due to anthropogenic extinctions of reef corals has never been explored using a fossil-calibrated phylogeny. Given that reef corals are facing a multitude of anthropogenic impacts with well-documented negative effects (Hughes et al. 2003; Bruno and Selig 2007; Bruno et al. 2007; Pandolfi et al. 2011; De'ath et al. 2012), knowledge of how much evolutionary history may be at risk due to individual threats should be useful for prioritizing conservation efforts, especially given the large variation in predicted PD loss across the different threat categories documented here. For example, bleaching and disease are among the well-recognized threats to reef corals (Aronson and Precht 2001; Willis et al. 2004; Selig et al. 2006; Bruno et al. 2007; Miller et al. 2009), and our results show that future species extinctions resulting from these threats would erase a greater part of the evolutionary history of this group compared to losses from other impacts.

## Conclusion

Anthropogenic impacts have the potential to obliterate a substantial amount of existing evolutionary history of reef corals, but the magnitude of loss varies considerably among different threat categories, with bleaching and disease emerging as the two biggest threats to the phylogenetic diversity of this group. Phylogenetic clustering of extinctions is a useful but incomplete predictor of the potential for future PD loss of corals; more comprehensive predictions also require knowledge of actual branch lengths and patterns of divergence. We suspect that this is likely to be true for many other groups as well. More generally, because tree shapes of living clades reflect a complex set of historical contingencies that are unique to individual taxa, patterns of PD loss due to anthropogenic extinctions are likely to be group specific. Thus, estimates of PD loss under specific threats using better resolved and calibrated trees are urgently needed to better understand how we, as a species, are pruning the rest of the tree of life.

## Supporting Information

**Table S1.** Scleractinian coral species used in the phylogenetic analysis.
