# Supplementary material for: Anthropogenic extinction threats and future loss of evolutionary history in reef corals
Source: Ecol Evol. 2013 Mar 18;3(5):1184–93. doi: 10.1002/ece3.527 (PMC3678474; doi:10.1002/ece3.527)
Supplement: Supplementary file 1 [file ece30003-1184-SD1.pdf]

Table S1. Scleractinian coral species used in the phylogenetic analysis.

Family	Genus/Species	Reef	Red List	Molecular sources	Morphological sources	Remarks
Acroporidae	*Acropora abrolhosensis*	Yes	VU	ND5: EU533959	[S1]	
Acroporidae	*Acropora abrotanoides*	Yes	LC	CTR: FJ899068	[S1]	
Acroporidae	*Acropora aculeus*	Yes	VU		[S1]	
Acroporidae	*Acropora acuminata*	Yes	VU	ND5: EU533969	[S1]	
Acroporidae	*Acropora akajimensis*	Yes	DD		[S1]	Junior synonym of A. donei [S1]
Acroporidae	*Acropora anthocercis*	Yes	VU	ND5: EU533970	[S1]	
Acroporidae	*Acropora appressa*	Yes	NT		[S1]	
Acroporidae	*Acropora arabensis*	Yes	NT		[S1]	Sister species to A. valida [S1]
Acroporidae	*Acropora aspera*	Yes	VU	CTR: EU918267; CYB: FJ391987	[S1]	
Acroporidae	*Acropora austera*	Yes	NT	CTR: EU918228; CYB: FJ391989	[S1]	
Acroporidae	*Acropora awi*	Yes	VU		[S1]	
Acroporidae	*Acropora batunai*	Yes	VU	CTR: EU918250; ND5: EU533971	[S1]	
Acroporidae	*Acropora bifurcata*	Yes	DD		[S1]	Junior synonym of A. hyacinthus [S1]
Acroporidae	*Acropora branchi*	Yes	DD		[S1]	
Acroporidae	*Acropora bushyensis*	Yes	LC	ND5: EU533975	[S1]	
Acroporidae	*Acropora cardenae*	Yes	DD		[S1]	
Acroporidae	*Acropora carduus*	Yes	NT	ND5: EU533976	[S1]	
Acroporidae	*Acropora caroliniana*	Yes	VU	CTR: EU918274	[S1]	
Acroporidae	*Acropora cerealis*	Yes	LC	CTR: EU918248; CYB: AF099652; ND5: EU533979	[S1]	
Acroporidae	*Acropora cervicornis*	Yes	CR	12S: EF597094; COI: AY451340; CTR: EU918257; CYB: AF099654; ND5: EU533960	[S1]	
Acroporidae	*Acropora chesterfieldensis*	Yes	LC	CTR: EU918262; ND5: EU533981	[S1]	
Acroporidae	*Acropora clathrata*	Yes	LC		[S1]	

Family	Genus/Species	Reef	Red List	Molecular sources	Morphological sources	Remarks
Acroporidae	*Acropora convexa*	Yes	DD		[S1]	Junior synonym of A. millepora [S1]
Acroporidae	*Acropora cophodactyla*	Yes	DD		[S1]	Junior synonym of A. humilis [S1]
Acroporidae	*Acropora copiosa*	Yes	DD		[S1]	Junior synonym of A. muricata [S1]
Acroporidae	*Acropora cytherea*	Yes	LC	12S: AF333054; 16S: L75995; CTR: AY083876; CYB: FJ391995	[S1]	
Acroporidae	*Acropora dendrum*	Yes	VU	ND5: EU533983	[S1]	Sister species to A. loisetteae [S1]
Acroporidae	*Acropora derawanensis*	Yes	VU	CTR: EU918263; ND5: EU533984	[S1]	
Acroporidae	*Acropora desalwii*	Yes	VU		[S1]	
Acroporidae	*Acropora digitifera*	Yes	NT	12S: AF333051; AT6: AB033199; CTR: EU918261; CYB: AB033184	[S1]	
Acroporidae	*Acropora divaricata*	Yes	NT	CTR: AY026432; ND5: EU533985	[S1]	
Acroporidae	*Acropora donei*	Yes	VU	AT6: AB033195; CYB: AB033180	[S1]	
Acroporidae	*Acropora downingi*	Yes	LC		[S1]	Sister species to A. polystoma [S1]
Acroporidae	*Acropora echinata*	Yes	VU	CYB: FJ391985; ND5: EU533986	[S1]	
Acroporidae	*Acropora efflorescens*	Yes	DD		[S1]	Junior synonym of A. cytherea [S1]
Acroporidae	*Acropora elegans*	Yes	VU	ND5: EU533990	[S1]	
Acroporidae	*Acropora elegantula*	Yes	DD		[S1]	
Acroporidae	*Acropora elseyi*	Yes	LC	ND5: EU533991	[S1]	
Acroporidae	*Acropora exquisita*	Yes	DD		[S1]	
Acroporidae	*Acropora fastigata*	Yes	DD		[S1]	
Acroporidae	*Acropora fenneri*	Yes	DD		[S1]	
Acroporidae	*Acropora filiformis*	Yes	DD		[S1]	
Acroporidae	*Acropora florida*	Yes	NT	AT6: AB033197; CTR: AY026435; CYB: AB033182; ND5: EU533993	[S1]	

Family	Genus/Species	Reef	Red List	Molecular sources	Morphological sources	Remarks
Acroporidae	*Acropora forskali*	Yes	DD		[S1]	
Acroporidae	*Acropora gemmifera*	Yes	LC	AT6: AB033198; CTR: EU918277; CYB: AB033183	[S1]	
Acroporidae	*Acropora glauca*	Yes	NT		[S1]	
Acroporidae	*Acropora globiceps*	Yes	VU	CTR: EF206433	[S1]	Sister species to A. humilis [S1]
Acroporidae	*Acropora gomezi*	Yes	DD		[S1]	
Acroporidae	*Acropora grandis*	Yes	LC	ND5: EU533994	[S1]	
Acroporidae	*Acropora granulosa*	Yes	NT	ND5: EU533995	[S1]	
Acroporidae	*Acropora haimei*	Yes	DD		[S1]	
Acroporidae	*Acropora halmaherae*	Yes	DD		[S1]	
Acroporidae	*Acropora hemprichii*	Yes	VU	16S: AF550359	[S1]	Sister species to A. austera [S1]
Acroporidae	*Acropora hoeksemai*	Yes	VU		[S1]	
Acroporidae	*Acropora horrida*	Yes	VU	ND5: EU533998	[S1]	
Acroporidae	*Acropora humilis*	Yes	NT	16S: L75996; CTR: EU918282; CYB: EF363316	[S1]	
Acroporidae	*Acropora hyacinthus*	Yes	NT	12S: AF333053; CTR: AY083877; CYB: FJ391988; ND5: EU534002	[S1]	
Acroporidae	*Acropora indonesia*	Yes	VU	ND5: EU534003	[S1]	
Acroporidae	*Acropora inermis*	Yes	DD		[S1]	Junior synonym of A. horrida [S1]
Acroporidae	*Acropora insignis*	Yes	DD		[S1]	
Acroporidae	*Acropora intermedia*	Yes	LC	CTR: AY026451; ND5: EU533965	[S1]	Senior synonym of A. nobilis [S1]
Acroporidae	*Acropora irregularis*	Yes	DD		[S1]	Junior synonym of A. abrotanoides [S1]
Acroporidae	*Acropora jacquelineae*	Yes	VU	CTR: EU918284; ND5: EU534012	[S1]	
Acroporidae	*Acropora japonica*	Yes	DD		[S1]	
Acroporidae	*Acropora khayranensis*	Yes	DD		[S1]	
Acroporidae	*Acropora kimbeensis*	Yes	VU	CTR: EU918214	[S1]	
Acroporidae	*Acropora kirstyae*	Yes	VU	CTR: EU918215; ND5: EU534015	[S1]	

Family	Genus/Species	Reef	Red List	Molecular sources	Morphological sources	Remarks
Acroporidae	*Acropora kosurini*	Yes	VU		[S1]	
Acroporidae	*Acropora lamarcki*	Yes	DD		[S1]	
Acroporidae	*Acropora latistella*	Yes	LC	CTR: AY026443; CYB: AF099656	[S1]	
Acroporidae	*Acropora lianae*	Yes	DD		[S1]	
Acroporidae	*Acropora listeri*	Yes	VU	ND5: EU533966	[S1]	
Acroporidae	*Acropora loisetteae*	Yes	VU	CTR: EU918222	[S1]	
Acroporidae	*Acropora lokani*	Yes	VU	CTR: EU918270	[S1]	
Acroporidae	*Acropora longicyathus*	Yes	LC	CTR: EU918220; ND5: EU534017	[S1]	
Acroporidae	*Acropora loripes*	Yes	NT	CTR: EU918227; ND5: EU534020	[S1]	
Acroporidae	*Acropora lovelli*	Yes	VU		[S1]	
Acroporidae	*Acropora lutkeni*	Yes	NT	ND5: EU534023	[S1]	
Acroporidae	*Acropora macrostoma*	Yes	DD		[S1]	Junior synonym of A. tenuis [S1]
Acroporidae	*Acropora maryae*	Yes	DD		[S1]	
Acroporidae	*Acropora massawensis*	Yes	DD		[S1]	Junior synonym of A. polystoma [S1]
Acroporidae	*Acropora microclados*	Yes	VU	ND5: EU534024	[S1]	
Acroporidae	*Acropora microphthalma*	Yes	LC	CTR: EU918203; CYB: FJ391986; ND5: EU534026	[S1]	
Acroporidae	*Acropora millepora*	Yes	NT	CTR: EU918207; CYB: AF099653; ND5: EU534029	[S1]	
Acroporidae	*Acropora minuta*	Yes	DD		[S1]	
Acroporidae	*Acropora mirabilis*	Yes	DD		[S1]	
Acroporidae	*Acropora monticulosa*	Yes	NT	CTR: EF206487	[S1]	
Acroporidae	*Acropora multiacuta*	Yes	VU	CTR: EF206546	[S1]	
Acroporidae	*Acropora muricata*	Yes	NT	12S: AF177042; CYB: AF099651	[S1]	Type species of Acropora [S1]
Acroporidae	*Acropora nana*	Yes	NT	ND5: EU534031	[S1]	

Family	Genus/Species	Reef	Red List	Molecular sources	Morphological sources	Remarks
Acroporidae	*Acropora nasuta*	Yes	NT	AT6: AB033200; CTR: EU918229; CYB: AB033185; ND5: EU534032	[S1]	
Acroporidae	*Acropora natalensis*	Yes	DD		[S1]	Junior synonym of A. solitaryensis [S1]
Acroporidae	*Acropora navini*	Yes	DD		[S1]	
Acroporidae	*Acropora ocellata*	Yes	DD		[S1]	Senior synonym of A. lutkeni [S1]
Acroporidae	*Acropora orbicularis*	Yes	DD		[S1]	Junior synonym of A. clathrata [S1]
Acroporidae	*Acropora pagoensis*	Yes	DD		[S1]	
Acroporidae	*Acropora palmata*	Yes	CR	12S: EF597092; COI: AB441246; CTR: AF507217; CYB: AB441331; ND5: EU533962	[S1]	
Acroporidae	*Acropora palmerae*	Yes	VU		[S1]	
Acroporidae	*Acropora paniculata*	Yes	VU		[S1]	
Acroporidae	*Acropora papillare*	Yes	VU	CTR: EU918211	[S1]	
Acroporidae	*Acropora paragemmifera*	Yes	DD		[S1]	
Acroporidae	*Acropora parahemprichii*	Yes	DD		[S1]	
Acroporidae	*Acropora parapharaonis*	Yes	DD		[S1]	
Acroporidae	*Acropora parilis*	Yes	DD		[S1]	Junior synonym of A. horrida [S1]
Acroporidae	*Acropora pectinatus*	Yes	DD		[S1]	
Acroporidae	*Acropora pharaonis*	Yes	VU		[S1]	
Acroporidae	*Acropora pichoni*	Yes	NT	CTR: EU918206; ND5: EU534033	[S1]	Sister species to A. elegans [S1]
Acroporidae	*Acropora pinguis*	Yes	DD		[S1]	Junior synonym of A. robusta [S1]
Acroporidae	*Acropora plana*	Yes	DD		[S1]	Junior synonym of A. tenuis [S1]
Acroporidae	*Acropora plantaginea*	Yes	DD		[S1]	
Acroporidae	*Acropora plumosa*	Yes	VU		[S1]	
Acroporidae	*Acropora polystoma*	Yes	VU	ND5: EU533964	[S1]	
Acroporidae	*Acropora prostrata*	Yes	DD		[S1]	Junior synonym of A. millepora [S1]
Acroporidae	*Acropora proximalis*	Yes	DD		[S1]	
Acroporidae	*Acropora pruinosa*	Yes	DD		[S1]	

Family	Genus/Species	Reef	Red List	Molecular sources	Morphological sources	Remarks
Acroporidae	*Acropora pulchra*	Yes	LC	CTR: EU918230; ND5: EU533967	[S1]	
Acroporidae	*Acropora rambleri*	Yes	DD		[S1]	Junior synonym of A. speciosa [S1]
Acroporidae	*Acropora retusa*	Yes	VU	CTR: EF206535	[S1]	
Acroporidae	*Acropora ridzwani*	Yes	DD		[S1]	
Acroporidae	*Acropora robusta*	Yes	LC	CTR: FJ899064	[S1]	
Acroporidae	*Acropora rongelapensis*	Yes	DD	CTR: EU918210	[S1]	
Acroporidae	*Acropora rosaria*	Yes	DD		[S1]	
Acroporidae	*Acropora roseni*	Yes	EN		[S1]	
Acroporidae	*Acropora rudis*	Yes	EN		[S1]	
Acroporidae	*Acropora rufus*	Yes	DD		[S1]	
Acroporidae	*Acropora russelli*	Yes	VU		[S1]	
Acroporidae	*Acropora samoensis*	Yes	LC	CTR: AY364095; CYB: FJ391994	[S1]	
Acroporidae	*Acropora sarmentosa*	Yes	LC	CTR: AY026455; ND5: EU534034	[S1]	
Acroporidae	*Acropora scherzeriana*	Yes	DD		[S1]	Junior synonym of A. gemmifera [S1]
Acroporidae	*Acropora schmitti*	Yes	DD		[S1]	Junior synonym of A. digitifera [S1]
Acroporidae	*Acropora secale*	Yes	NT		[S1]	
Acroporidae	*Acropora sekiseiensis*	Yes	DD		[S1]	Junior synonym of A. horrida [S1]
Acroporidae	*Acropora selago*	Yes	NT	CTR: AB361179; ND5: EU534035	[S1]	
Acroporidae	*Acropora seriata*	Yes	DD		[S1]	
Acroporidae	*Acropora simplex*	Yes	VU		[S1]	
Acroporidae	*Acropora solitaryensis*	Yes	VU	ND5: EU534039	[S1]	
Acroporidae	*Acropora spathulata*	Yes	LC	CTR: EU918209; ND5: EU534040	[S1]	
Acroporidae	*Acropora speciosa*	Yes	VU	CTR: EU918245	[S1]	
Acroporidae	*Acropora spicifera*	Yes	VU	CTR: AY083881; ND5: EU534041	[S1]	
Acroporidae	*Acropora squarrosa*	Yes	LC		[S1]	Sister species to A. loripes [S1]
Acroporidae	*Acropora stoddarti*	Yes	DD		[S1]	Junior synonym of A. divaricata [S1]
Acroporidae	*Acropora striata*	Yes	VU		[S1]	

Family	Genus/Species	Reef	Red List	Molecular sources	Morphological sources	Remarks
Acroporidae	*Acropora subglabra*	Yes	LC	ND5: EU534042	[S1]	
Acroporidae	*Acropora subulata*	Yes	LC		[S1]	
Acroporidae	*Acropora suharsonoi*	Yes	EN		[S1]	
Acroporidae	*Acropora sukarnoi*	Yes	DD		[S1]	
Acroporidae	*Acropora tanegashimensis*	Yes	DD		[S1]	Sister species to A. hyacinthus [S1]
Acroporidae	*Acropora tenella*	Yes	VU	CTR: EU918240	[S1]	
Acroporidae	*Acropora tenuis*	Yes	NT	12S: AF338425; 16S: AF338425; AT6: AF338425; COI: AF338425; CTR: AF338425; CYB: AF338425; ND5: AF338425	[S1]	
Acroporidae	*Acropora teres*	Yes	DD		[S1]	
Acroporidae	*Acropora tizardi*	Yes	DD		[S1]	Junior synonym of A. cerealis [S1]
Acroporidae	*Acropora torihalimeda*	Yes	DD		[S1]	
Acroporidae	*Acropora torresiana*	Yes	DD		[S1]	
Acroporidae	*Acropora tortuosa*	Yes	LC	CTR: EU918238	[S1]	
Acroporidae	*Acropora tumida*	Yes	DD		[S1]	Junior synonym of A. valida [S1]
Acroporidae	*Acropora turaki*	Yes	VU		[S1]	
Acroporidae	*Acropora tutuilensis*	Yes	DD		[S1]	Junior synonym of A. abrotanoides [S1]
Acroporidae	*Acropora valenciennesi*	Yes	LC		[S1]	
Acroporidae	*Acropora valida*	Yes	LC	CTR: EU918235; CYB: AF099658; ND5: EU534047	[S1]	
Acroporidae	*Acropora variabilis*	Yes	DD		[S1]	Junior synonym of A. valida [S1]
Acroporidae	*Acropora variolosa*	Yes	LC		[S1]	Sister species to A. rudis [S1]
Acroporidae	*Acropora vaughani*	Yes	VU	CTR: EU918224	[S1]	
Acroporidae	*Acropora vermiculata*	Yes	DD		[S1]	Junior synonym of A. sarmentosa [S1]
Acroporidae	*Acropora verweyi*	Yes	VU		[S1]	
Acroporidae	*Acropora walindii*	Yes	VU	CTR: EU918234	[S1]	Sister species to A. tenella [S1]
Acroporidae	*Acropora wallaceae*	Yes	DD		[S1]	Junior synonym of A. samoensis [S1]
Acroporidae	*Acropora willisae*	Yes	VU		[S1]	

Family	Genus/Species	Reef	Red List	Molecular sources	Morphological sources	Remarks
Acroporidae	*Acropora yongei*	Yes	LC	ND5: EU534048	[S1]	
Acroporidae	*Anacropora forbesi*	Yes	LC	COI: AB441251; CYB: AB441336	[S1]	
Acroporidae	*Anacropora matthai*	Yes	VU	12S: AY903295; 16S: AY903295; AT6: AY903295; COI: AY903295; CTR: AY903295; CYB: AY903295; ND5: AY903295	[S1]	
Acroporidae	*Anacropora pillai*	Yes	DD		[S1]	
Acroporidae	*Anacropora puertogalerae*	Yes	VU		[S1]	
Acroporidae	*Anacropora reticulata*	Yes	VU		[S1]	
Acroporidae	*Anacropora spinosa*	Yes	EN		[S1]	
Acroporidae	*Anacropora spumosa*	Yes	DD		[S1]	
Acroporidae	*Astreopora acroporina*	Yes	DD		[S1]	New species [S2]
Acroporidae	*Astreopora cenderawasih*	Yes	DD		[S1]	New species [S2]
Acroporidae	*Astreopora cucullata*	Yes	VU		[S1]	
Acroporidae	*Astreopora eliptica*	Yes	DD		[S1]	
Acroporidae	*Astreopora expansa*	Yes	NT		[S1]	
Acroporidae	*Astreopora gracilis*	Yes	LC		[S1]	
Acroporidae	*Astreopora incrustans*	Yes	VU		[S1]	
Acroporidae	*Astreopora listeri*	Yes	LC		[S1]	
Acroporidae	*Astreopora macrostoma*	Yes	NT		[S1]	
Acroporidae	*Astreopora montiporina*	Yes	DD		[S1]	New species [S2]
Acroporidae	*Astreopora moretonensis*	Yes	VU		[S1]	
Acroporidae	*Astreopora myriophthalma*	Yes	LC	12S: AF177046; AT6: AB033186; COI: AB441253; CYB: AB441338	[S1]	
Acroporidae	*Astreopora ocellata*	Yes	LC		[S1]	
Acroporidae	*Astreopora randalli*	Yes	LC		[S1]	
Acroporidae	*Astreopora scabra*	Yes	LC		[S1]	
Acroporidae	*Astreopora suggesta*	Yes	LC		[S1]	

Family	Genus/Species	Reef	Red List	Molecular sources	Morphological sources	Remarks
Acroporidae	*Enigmopora darveliensis*	Yes	DD			Closest to Acropora (prior to elevation of Isopora) [S3]
Acroporidae	*Isopora brueggemanni*	Yes	VU	12S: AF333048; AT6: AB033193; COI: AB441247; CYB: AB441332; ND5: EU534004	[S1]	
Acroporidae	*Isopora crateriformis*	Yes	VU		[S1]	
Acroporidae	*Isopora cuneata*	Yes	VU	12S: AF333049; CTR: AY026429; ND5: EU534006	[S1]	
Acroporidae	*Isopora cylindrica*	Yes	DD		[S1]	
Acroporidae	*Isopora elizabethensis*	Yes	DD		[S1]	
Acroporidae	*Isopora meridiana*	Yes	DD		[S1]	Junior synonym of Acropora brueggemanni [S1]
Acroporidae	*Isopora palifera*	Yes	NT	12S: AF177044; 16S: AF265593; AT6: AB033194; COI: AB441248; CYB: AB441333; ND5: EU534010	[S1]	
Acroporidae	*Isopora togianensis*	Yes	EN	12S: AF333050; COI: AB441249; CYB: AB441334; ND5: EU534008	[S1]	Morphological coding updated [S4]
Acroporidae	*Montipora aequituberculata*	Yes	LC	12S: AF333045; AT6: AB033187; CTR: AY313548; CYB: AB033172	[S1]	
Acroporidae	*Montipora altasepta*	Yes	VU	AT6: AB033190; CTR: AY313572; CYB: AB033175	[S1]	
Acroporidae	*Montipora angulata*	Yes	VU	CTR: AY313563	[S1]	
Acroporidae	*Montipora aspergillus*	Yes	DD		[S1]	
Acroporidae	*Montipora australiensis*	Yes	VU		[S1]	

Family	Genus/Species	Reef	Red List	Molecular sources	Morphological sources	Remarks
Acroporidae	*Montipora cactus*	Yes	VU	12S: AY903296; 16S: AY903296; AT6: AY903296; COI: AY903296; CTR: AY903296; CYB: AY903296; ND5: AY903296	[S1]	
Acroporidae	*Montipora calcarea*	Yes	VU		[S1]	
Acroporidae	*Montipora caliculata*	Yes	VU		[S1]	
Acroporidae	*Montipora capitata*	Yes	NT	16S: HQ246709; AT6: HQ246686; COI: HQ246613; CTR: HQ246520; CYB: HQ246516	[S1]	
Acroporidae	*Montipora capricornis*	Yes	VU	CTR: AY313583	[S1]	
Acroporidae	*Montipora cebuensis*	Yes	VU		[S1]	
Acroporidae	*Montipora circumvallata*	Yes	LC	16S: AF550368	[S1]	
Acroporidae	*Montipora cocosensis*	Yes	VU		[S1]	
Acroporidae	*Montipora confusa*	Yes	NT	CTR: AY313551	[S1]	
Acroporidae	*Montipora corbettensis*	Yes	VU		[S1]	
Acroporidae	*Montipora crassituberculata*	Yes	VU		[S1]	
Acroporidae	*Montipora cryptus*	Yes	NT		[S1]	
Acroporidae	*Montipora danae*	Yes	LC	CTR: AY313549	[S1]	
Acroporidae	*Montipora delicatula*	Yes	VU	CTR: AY313566	[S1]	
Acroporidae	*Montipora digitata*	Yes	LC	12S: AF177045; 16S: L75993; AT6: AB033188; CTR: AY313579; CYB: AB033173	[S1]	
Acroporidae	*Montipora dilatata*	Yes	EN	16S: HQ246702; AT6: HQ246678; COI: HQ246605; CTR: HQ246554; CYB: HQ246508	[S1]	
Acroporidae	*Montipora echinata*	Yes	DD		[S1]	

Family	Genus/Species	Reef	Red List	Molecular sources	Morphological sources	Remarks
Acroporidae	*Montipora efflorescens*	Yes	NT	AT6: AB033189; CYB: AB033174	[S1]	
Acroporidae	*Montipora effusa*	Yes	NT		[S1]	
Acroporidae	*Montipora flabellata*	Yes	VU	16S: HQ246698; AT6: HQ246674; COI: HQ246601; CTR: HQ246561; CYB: HQ246504	[S1]	
Acroporidae	*Montipora florida*	Yes	VU	CTR: AY313562	[S1]	
Acroporidae	*Montipora floweri*	Yes	LC		[S1]	
Acroporidae	*Montipora foliosa*	Yes	NT	CYB: FJ392003	[S1]	
Acroporidae	*Montipora foveolata*	Yes	NT		[S1]	
Acroporidae	*Montipora friabilis*	Yes	VU		[S1]	
Acroporidae	*Montipora gaimardi*	Yes	VU	CTR: AY313565	[S1]	
Acroporidae	*Montipora grisea*	Yes	LC		[S1]	
Acroporidae	*Montipora hemispherica*	Yes	DD		[S1]	
Acroporidae	*Montipora hirsuta*	Yes	NT		[S1]	
Acroporidae	*Montipora hispida*	Yes	LC	CTR: AY313553; CYB: FJ392005	[S1]	
Acroporidae	*Montipora hodgsoni*	Yes	VU		[S1]	
Acroporidae	*Montipora hoffmeisteri*	Yes	LC	CTR: AY313580	[S1]	
Acroporidae	*Montipora incrassata*	Yes	NT	16S: HQ246710; AT6: HQ246687; COI: HQ246614; CTR: HQ246593; CYB: HQ246517	[S1]	Montipora cf. incrassata in GenBank
Acroporidae	*Montipora informis*	Yes	LC		[S1]	
Acroporidae	*Montipora kellyi*	Yes	DD		[S1]	
Acroporidae	*Montipora lobulata*	Yes	VU		[S1]	
Acroporidae	*Montipora mactanensis*	Yes	VU		[S1]	
Acroporidae	*Montipora malampaya*	Yes	VU		[S1]	
Acroporidae	*Montipora meandrina*	Yes	VU		[S1]	
Acroporidae	*Montipora millepora*	Yes	LC		[S1]	
Acroporidae	*Montipora mollis*	Yes	LC	CTR: AY313552	[S1]	

Family	Genus/Species	Reef	Red List	Molecular sources	Morphological sources	Remarks
Acroporidae	*Montipora monasteriata*	Yes	LC		[S1]	
Acroporidae	*Montipora niugini*	Yes	NT		[S1]	
Acroporidae	*Montipora nodosa*	Yes	NT		[S1]	
Acroporidae	*Montipora orientalis*	Yes	VU		[S1]	
Acroporidae	*Montipora pachytuberculata*	Yes	DD		[S1]	
Acroporidae	*Montipora palawanensis*	Yes	NT		[S1]	
Acroporidae	*Montipora patula*	Yes	VU	16S: HQ246691; AT6: HQ246667; COI: HQ246595; CTR: HQ246573; CYB: HQ246497	[S1]	
Acroporidae	*Montipora peltiformis*	Yes	NT	CTR: AY313550	[S1]	
Acroporidae	*Montipora porites*	Yes	NT		[S1]	
Acroporidae	*Montipora samarensis*	Yes	VU		[S1]	
Acroporidae	*Montipora saudii*	Yes	NT		[S1]	
Acroporidae	*Montipora setosa*	Yes	EN		[S1]	
Acroporidae	*Montipora spongiosa*	Yes	LC		[S1]	
Acroporidae	*Montipora spongodes*	Yes	LC	CTR: AY313547	[S1]	
Acroporidae	*Montipora spumosa*	Yes	LC		[S1]	
Acroporidae	*Montipora stellata*	Yes	LC	CTR: AY313587	[S1]	
Acroporidae	*Montipora stilosa*	Yes	VU		[S1]	
Acroporidae	*Montipora taiwanensis*	Yes	DD		[S1]	
Acroporidae	*Montipora tuberculosa*	Yes	LC		[S1]	
Acroporidae	*Montipora turgescens*	Yes	LC	16S: HQ246704; AT6: HQ246681; COI: HQ246608; CTR: HQ246564; CYB: HQ246511	[S1]	Montipora cf. turgescens in GenBank
Acroporidae	*Montipora turtlensis*	Yes	VU	CTR: AY313574	[S1]	
Acroporidae	*Montipora undata*	Yes	NT	CTR: AY313569	[S1]	
Acroporidae	*Montipora vaughani*	Yes	DD		[S1]	
Acroporidae	*Montipora venosa*	Yes	NT		[S1]	

Family	Genus/Species	Reef	Red List	Molecular sources	Morphological sources	Remarks
Acroporidae	*Montipora verrilli*	Yes	DD	16S: HQ246695; AT6: HQ246671; COI: HQ246598; CTR: HQ246582; CYB: HQ246501	[S1]	
Acroporidae	*Montipora verrucosa*	Yes	LC	12S: EF597090; CTR: AY313584	[S1]	
Acroporidae	*Montipora verruculosus*	Yes	VU		[S1]	
Acroporidae	*Montipora vietnamensis*	Yes	VU		[S1]	
Agariciidae	*Agaricia agaricites*	Yes	LC	12S: EF597079; COI: AY451366	[S5]	
Agariciidae	*Agaricia fragilis*	Yes	DD	12S: EF597077	[S5]	
Agariciidae	*Agaricia grahamae*	Yes	LC	12S: EF597078		
Agariciidae	*Agaricia humilis*	Yes	LC	12S: DQ643831; 16S: DQ643831; AT6: DQ643831; COI: DQ643831; CYB: DQ643831; ND5: DQ643831		
Agariciidae	*Agaricia lamarcki*	Yes	VU	12S: EF597076; COI: AY451369		
Agariciidae	*Agaricia tenuifolia*	Yes	NT	12S: EF597081; COI: AY451370		
Agariciidae	*Agaricia undata*	Yes	DD	12S: EF597075		
Agariciidae	*Coeloseris mayeri*	Yes	LC			Closest to Pavona [S6]
Agariciidae	*Gardineroseris planulata*	Yes	LC	12S: EF597084; COI: AB441218; CYB: AB441303		
Agariciidae	*Helioseris cucullata*	Yes	LC	COI: AB441220; CYB: AB441305	[S5]	
Agariciidae	*Leptoseris amitoriensis*	Yes	NT			Leptoseris monophyly assumed; closest to L. papyracea [S7]
Agariciidae	*Leptoseris cailleti*	Yes	LC			Leptoseris monophyly assumed; closest to L. papyracea [S8]
Agariciidae	*Leptoseris explanata*	Yes	LC			Leptoseris monophyly assumed

Family	Genus/Species	Reef	Red List	Molecular sources	Morphological sources	Remarks
Agariciidae	*Leptoseris foliosa*	Yes	LC			Leptoseris monophyly assumed; closest to L. mycetoseroides [S6]
Agariciidae	*Leptoseris gardineri*	Yes	LC			Leptoseris monophyly assumed; closest to L. papyracea [S6]
Agariciidae	*Leptoseris hawaiiensis*	Yes	LC			Leptoseris monophyly assumed; closest to L. scabra [S6]
Agariciidae	*Leptoseris incrustans*	Yes	VU	16S: L76012		
Agariciidae	*Leptoseris mycetoseroides*	Yes	LC			Leptoseris monophyly assumed
Agariciidae	*Leptoseris papyracea*	Yes	LC			Leptoseris monophyly assumed
Agariciidae	*Leptoseris scabra*	Yes	LC			Leptoseris monophyly assumed
Agariciidae	*Leptoseris solida*	Yes	LC			Leptoseris monophyly assumed; closest to L. scabra [S8]
Agariciidae	*Leptoseris striata*	Yes	NT			Leptoseris monophyly assumed; closest to L. scabra [S9]
Agariciidae	*Leptoseris tubulifera*	Yes	LC			Leptoseris monophyly assumed
Agariciidae	*Leptoseris yabei*	Yes	VU			Leptoseris monophyly assumed; closest to L. mycetoseroides [S6]
Agariciidae	*Pachyseris foliosa*	Yes	LC			Pachyseris monophyly assumed; closest to P. involuta [S7]
Agariciidae	*Pachyseris gemmae*	Yes	NT			Pachyseris monophyly assumed; closest to P. rugosa & P. speciosa [S8]
Agariciidae	*Pachyseris involuta*	Yes	VU			Pachyseris monophyly assumed
Agariciidae	*Pachyseris rugosa*	Yes	VU			Pachyseris monophyly assumed
Agariciidae	*Pachyseris speciosa*	Yes	LC	COI: AB441222; CYB: AB441307		
Agariciidae	*Pavona bipartita*	Yes	VU		[S10]	
Agariciidae	*Pavona cactus*	Yes	VU	16S: AF550370; COI: AB441217; CYB: AB441302	[S10]	
Agariciidae	*Pavona chiriquiensis*	Yes	LC		[S10]	

Family	Genus/Species	Reef	Red List	Molecular sources	Morphological sources	Remarks
Agariciidae	*Pavona clavus*	Yes	LC	12S: DQ643836; 16S: DQ643836; AT6: DQ643836; COI: DQ643836; CYB: DQ643836; ND5: DQ643836	[S10]	
Agariciidae	*Pavona danai*	Yes	VU		[S10]	
Agariciidae	*Pavona decussata*	Yes	VU		[S10]	
Agariciidae	*Pavona diffluens*	Yes	VU		[S10]	
Agariciidae	*Pavona duerdeni*	Yes	LC		[S10]	
Agariciidae	*Pavona explanulata*	Yes	LC		[S10]	
Agariciidae	*Pavona frondifera*	Yes	LC	12S: AF333055	[S10]	
Agariciidae	*Pavona gigantea*	Yes	LC		[S10]	
Agariciidae	*Pavona maldivensis*	Yes	LC		[S10]	
Agariciidae	*Pavona minuta*	Yes	NT		[S10]	
Agariciidae	*Pavona varians*	Yes	LC	12S: EF597083; 16S: L76016	[S10]	
Agariciidae	*Pavona venosa*	Yes	VU		[S10]	
Agariciidae	*Pavona xarifae*	Yes	DD		[S10]	
Anthemiphyllidae	*Anthemiphyllia dentata*	No	N/A	COI: HM018603	[S10]	
Anthemiphyllidae	*Anthemiphyllia patera*	No	N/A	COI: HM018604	[S10]	
Anthemiphyllidae	*Anthemiphyllia spinifera*	No	N/A	16S: AF265596	[S10]	
Astrocoeniidae	*Madracis asanoi*	Yes	DD	COI: HM018656		
Astrocoeniidae	*Madracis asperula*	Yes	DD			Closest to M. asanoi [S8]
Astrocoeniidae	*Madracis auretenra*	Yes	LC	12S: EU400212; 16S: EU400212; AT6: EU400212; COI: EU400212; CYB: EU400212		Probably M. mirabilis in GenBank [S11]
Astrocoeniidae	*Madracis carmabi*	Yes	DD	12S: EF596980		
Astrocoeniidae	*Madracis decactis*	Yes	LC	12S: EF596982	[S5]	
Astrocoeniidae	*Madracis formosa*	Yes	LC	12S: EF596981		
Astrocoeniidae	*Madracis kirbyi*	Yes	LC			Closest to M. decactis [S8]
Astrocoeniidae	*Madracis pharensis*	Yes	LC	12S: EF596983		

Family	Genus/Species	Reef	Red List	Molecular sources	Morphological sources	Remarks
Astrocoeniidae	*Madracis senaria*	Yes	LC	12S: EF596979		
Astrocoeniidae	*Palauastrea ramosa*	Yes	NT			Closest to Madracis [S12]
Astrocoeniidae	*Stephanocoenia intersepta*	Yes	LC	12S: EF597072; COI: AB441228; CYB: AB441313	[S5]	Senior synonym of S. michelinii
Astrocoeniidae	*Stylocoeniella armata*	Yes	LC			Stylocoeniella monophyly assumed; closest to S. guentheri [S8]
Astrocoeniidae	*Stylocoeniella cocosensis*	Yes	VU			Stylocoeniella monophyly assumed
Astrocoeniidae	*Stylocoeniella guentheri*	Yes	LC	COI: AB441225; CYB: AB441310		
Astrocoeniidae	*Stylocoeniella muscosus*	Yes	DD			Stylocoeniella monophyly assumed
Caryophylliidae	*Caryophyllia ambrosia*	No	N/A	16S: AF550362	[S5,S10]	
Caryophylliidae	*Caryophyllia atlantica*	No	N/A	16S: FJ788113; COI: HM018613	[S10]	
Caryophylliidae	*Caryophyllia diomedeae*	No	N/A	16S: FJ788115; COI: HM018614	[S10]	
Caryophylliidae	*Caryophyllia grandis*	No	N/A	16S: FJ788117	[S10]	
Caryophylliidae	*Caryophyllia grayi*	No	N/A	16S: FJ788119; COI: HM018615	[S10]	
Caryophylliidae	*Caryophyllia inornata*	No	N/A	12S: EF597042; 16S: AF265599	[S10]	
Caryophylliidae	*Caryophyllia lamellifera*	No	N/A	16S: FJ788120; COI: HM018616	[S10]	
Caryophylliidae	*Caryophyllia planilamellata*	No	N/A	16S: FJ788121	[S10]	
Caryophylliidae	*Caryophyllia ralphae*	No	N/A	COI: HM018617	[S10]	
Caryophylliidae	*Caryophyllia rugosa*	No	N/A	16S: FJ788123; COI: HM018618	[S10]	
Caryophylliidae	*Caryophyllia scobinosa*	No	N/A	16S: FJ788124	[S10]	
Caryophylliidae	*Caryophyllia transversalis*	No	N/A	16S: FJ788125	[S10]	
Caryophylliidae	*Caryophyllia unicristata*	No	N/A	16S: FJ788127	[S10]	
Caryophylliidae	*Ceratotrochus magnaghii*	No	N/A	16S: AF265597		
Caryophylliidae	*Conotrochus funicolumna*	No	N/A	COI: HM018621		
Caryophylliidae	*Crispatotrochus rugosus*	No	N/A	12S: EF597041; 16S: AF265600	[S10]	

Family	Genus/Species	Reef	Red List	Molecular sources	Morphological sources	Remarks
Caryophylliidae	*Dactylotrochus cervicornis*	No	N/A	COI: HM018624		
Caryophylliidae	*Dasmosmilia lymani*	No	N/A	16S: FJ788130; COI: HM018625	[S5]	Dasmosmilia cf. lymani in GenBank
Caryophylliidae	*Dasmosmilia variegata*	No	N/A		[S5]	Dasmosmilia monophyly assumed
Caryophylliidae	*Deltocyathus calcar*	No	N/A		[S5]	Deltocyathus monophyly assumed
Caryophylliidae	*Deltocyathus eccentricus*	No	N/A		[S5]	Deltocyathus monophyly assumed
Caryophylliidae	*Deltocyathus inusitatus*	No	N/A	COI: HM018626		
Caryophylliidae	*Deltocyathus italicus*	No	N/A		[S5]	
Caryophylliidae	*Deltocyathus magnificus*	No	N/A	COI: HM018627		
Caryophylliidae	*Deltocyathus ornatus*	No	N/A	COI: HM018628		
Caryophylliidae	*Deltocyathus rotulus*	No	N/A	COI: HM018629		
Caryophylliidae	*Deltocyathus sarsi*	No	N/A	COI: HM018630		
Caryophylliidae	*Deltocyathus suluensis*	No	N/A	COI: HM018631		
Caryophylliidae	*Desmophyllum dianthus*	No	N/A	12S: GQ868667; 16S: GQ868690		
Caryophylliidae	*Heterocyathus aequicostatus*	Yes	LC			Heterocyathus monophyly assumed
Caryophylliidae	*Heterocyathus alternatus*	Yes	LC			Heterocyathus monophyly assumed
Caryophylliidae	*Heterocyathus sulcatus*	Yes	LC	COI		Arrigoni and Benzoni (unpublished data)
Caryophylliidae	*Hoplangia durotrix*	No	N/A	12S: EF597064		
Caryophylliidae	*Lophelia pertusa*	No	N/A	12S: FR821799; 16S: FR821799; AT6: FR821799; COI: FR821799; CYB: FR821799		
Caryophylliidae	*Paracyathus pulchellus*	No	N/A	12S: EF597027; 16S: AF265603	[S10]	
Caryophylliidae	*Phyllangia americana*	No	N/A	12S: EF597022; 16S: AF265605	[S5,S10]	
Caryophylliidae	*Phyllangia papuensis*	No	N/A	COI: HM018660	[S10]	
Caryophylliidae	*Polycyathus muellerae*	No	N/A	12S: EF597026; 16S: AF265606	[S10]	
Caryophylliidae	*Premocyathus cornuformis*	No	N/A		[S5]	Revised from Caryophyllia cornuformis
Caryophylliidae	*Rhizosmilia maculata*	No	N/A	12S: EF597023; 16S: AF265602	[S5]	

Family	Genus/Species	Reef	Red List	Molecular sources	Morphological sources	Remarks
Caryophylliidae	*Rhizosmilia robusta*	No	N/A	COI: HM018664		
Caryophylliidae	*Solenosmilia variabilis*	No	N/A	16S: HM015348		
Caryophylliidae	*Stephanocyathus platypus*	No	N/A	16S: HM015352		
Caryophylliidae	*Stephanocyathus spiniger*	No	N/A	16S: HM015359; COI: HM018665		
Caryophylliidae	*Stephanocyathus weberianus*	No	N/A	16S: AF265594		
Caryophylliidae	*Tethocyathus virgatus*	No	N/A	16S: FJ788131		
Caryophylliidae	*Thalamophyllia gasti*	No	N/A	12S: EF597086; 16S: AF265590	[S10]	
Caryophylliidae	*Thalamophyllia riisei*	No	N/A	12S: EF597087	[S10]	
Caryophylliidae	*Trochocyathus efateensis*	No	N/A	16S: FJ788132; COI: HM018667		
Caryophylliidae	*Trochocyathus rhombocolumna*	No	N/A	COI: HM018668		
Caryophylliidae	*Vaughanella*	No	N/A	16S: AF265595	[S10]	
Dendrophylliidae	*Astroides calycularis*	No	N/A		[S13]	
Dendrophylliidae	*Balanophyllia (Eupsammia)*	No	N/A		[S10,S13]	Balanophyllia (Eupsammia)
Dendrophylliidae	*Balanophyllia cornu*	No	N/A	COI: HM018605	[S10,S13]	
Dendrophylliidae	*Balanophyllia desmophyllioides*	No	N/A	COI: HM018607	[S10,S13]	
Dendrophylliidae	*Balanophyllia elegans*	No	N/A	COI: DQ445805	[S10,S13]	
Dendrophylliidae	*Balanophyllia europaea*	Yes	DD		[S5,S10,S13]	
Dendrophylliidae	*Balanophyllia regia*	No	N/A	12S: EF597047; 16S: AF265587	[S10,S13]	
Dendrophylliidae	*Bathypsammia*	No	N/A		[S13]	
Dendrophylliidae	*Cladopsammia gracilis*	No	N/A	12S: EF597049; 16S: AF265588	[S13]	
Dendrophylliidae	*Dendrophyllia alternata*	No	N/A	16S: AF550366	[S13]	
Dendrophylliidae	*Dichopsammia granulosa*	No	N/A		[S13]	
Dendrophylliidae	*Duncanopsammia axifuga*	Yes	NT		[S13]	
Dendrophylliidae	*Eguchipsammia*	No	N/A		[S13]	
Dendrophylliidae	*Enallopsammia rostrata*	No	N/A	16S: U40294; COI: HM018632	[S10,S13]	
Dendrophylliidae	*Endopachys*	No	N/A		[S13]	
Dendrophylliidae	*Endopsammia*	No	N/A		[S13]	

Family	Genus/Species	Reef	Red List	Molecular sources	Morphological sources	Remarks
Dendrophylliidae	*Heteropsammia cochleata*	Yes	LC	12S: EF597050	[S13]	Heteropsammia sp. in GenBank
Dendrophylliidae	*Heteropsammia eupsammides*	Yes	NT		[S13]	
Dendrophylliidae	*Leptopsammia pruvoti*	No	N/A	12S: EF597068; 16S: AF265579	[S10,S13]	
Dendrophylliidae	*Notophyllia*	No	N/A		[S13]	
Dendrophylliidae	*Pourtalopsammia togata*	No	N/A		[S13]	
Dendrophylliidae	*Rhizopsammia*	No	N/A		[S10,S13]	
Dendrophylliidae	*Thecopsammia*	No	N/A		[S13]	
Dendrophylliidae	*Trochopsammia infundibulum*	No	N/A		[S13]	
Dendrophylliidae	*Tubastraea coccinea*	No	N/A	12S: EF597045; 16S: L76022; COI: DQ445806	[S10,S13]	
Dendrophylliidae	*Turbinaria bifrons*	Yes	VU		[S10,S13]	
Dendrophylliidae	*Turbinaria conspicua*	Yes	LC		[S10,S13]	
Dendrophylliidae	*Turbinaria crater*	Yes	DD		[S10,S13]	Previously excluded [S14]
Dendrophylliidae	*Turbinaria frondens*	Yes	LC		[S10,S13]	
Dendrophylliidae	*Turbinaria heronensis*	Yes	VU		[S10,S13]	
Dendrophylliidae	*Turbinaria irregularis*	Yes	LC		[S10,S13]	
Dendrophylliidae	*Turbinaria mesenterina*	Yes	VU		[S10,S13]	
Dendrophylliidae	*Turbinaria patula*	Yes	VU		[S10,S13]	
Dendrophylliidae	*Turbinaria peltata*	Yes	VU	12S: EF597044; COI: AB441240; CYB: AB441325	[S10,S13]	
Dendrophylliidae	*Turbinaria radicalis*	Yes	NT		[S10,S13]	
Dendrophylliidae	*Turbinaria reniformis*	Yes	VU		[S10,S13]	
Dendrophylliidae	*Turbinaria stellulata*	Yes	VU		[S10,S13]	
Euphylliidae	*Catalaphyllia jardinei*	Yes	VU	12S: EF596997; 16S: L76000	[S10]	
Euphylliidae	*Euphyllia ancora*	Yes	VU	12S: JF825139; 16S: JF825139; AT6: JF825139; COI: JF825139; CYB: JF825139; ND5: JF825139		

Family	Genus/Species	Reef	Red List	Molecular sources	Morphological sources	Remarks
Euphylliidae	*Euphyllia cristata*	Yes	VU			Closest to E. glabrescens [S15]
Euphylliidae	*Euphyllia divisa*	Yes	NT	COI: AB441203; CYB: AB441288		
Euphylliidae	*Euphyllia glabrescens*	Yes	NT	COI: AB441206; CYB: AB441291		
Euphylliidae	*Euphyllia paraancora*	Yes	VU			Closest to E. ancora & E. divisa [S16]
Euphylliidae	*Euphyllia paradivisa*	Yes	VU			Closest to E. glabrescens [S7]
Euphylliidae	*Euphyllia paraglabrescens*	Yes	VU			Closest to E. glabrescens [S16]
Euphylliidae	*Euphyllia yaeyamaensis*	Yes	NT			Closest to E. divisa [S8]
Euphylliidae	*Nemenzophyllia turbida*	Yes	VU			Closest to Plerogyra [S6]
Euphylliidae	*Physogyra lichtensteini*	Yes	VU	12S: EF597030; COI: AB289562; CYB: AB289564	[S5]	
Euphylliidae	*Plerogyra cauliformis*	Yes	DD			Plerogyra monophyly assumed; closest to P. simplex [S3]
Euphylliidae	*Plerogyra diabolotus*	Yes	DD			Plerogyra monophyly assumed
Euphylliidae	*Plerogyra discus*	Yes	VU			Plerogyra monophyly assumed; closest to P. sinuosa [S9]
Euphylliidae	*Plerogyra multilobata*	Yes	DD			Plerogyra monophyly assumed; closest to P. diabolotus [S3]
Euphylliidae	*Plerogyra simplex*	Yes	NT			Plerogyra monophyly assumed
Euphylliidae	*Plerogyra sinuosa*	Yes	NT	COI: HM018663		Plerogyra sp. in GenBank
Faviidae	*Australogyra zelli*	Yes	VU			Closest to Platygyra [S17]
Faviidae	*Barabattoia amicorum*	Yes	LC	COI: AB441193; CYB: AB441278		
Faviidae	*Barabattoia laddi*	Yes	VU			Clade VII-B monophyly shown [S18]; Barabattoia monophyly assumed
Faviidae	*Caulastraea connata*	Yes	VU			Closest to C. tumida [S8]
Faviidae	*Caulastraea curvata*	Yes	VU			Closest to C. furcata [S19]
Faviidae	*Caulastraea echinulata*	Yes	VU	COI: FJ345414		
Faviidae	*Caulastraea furcata*	Yes	LC	12S: EF597035; 16S: L75997; COI: AB117274; CYB: AB117355		

Family	Genus/Species	Reef	Red List	Molecular sources	Morphological sources	Remarks
Faviidae	*Caulastraea tumida*	Yes	NT	COI: HQ203249		
Faviidae	*Cladocora arbuscula*	Yes	LC	COI: AB117292; CYB: AB117377	[S5,S10]	
Faviidae	*Cladocora caespitosa*	Yes	DD	12S: EF597017; 16S: AF265612	[S10]	
Faviidae	*Cladocora debilis*	No	N/A		[S5,S10]	
Faviidae	*Colpophyllia amaranthus*	Yes	DD		[S20]	Previously excluded [S14]
Faviidae	*Colpophyllia breviserialis*	Yes	DD		[S20]	Previously excluded [S14]
Faviidae	*Colpophyllia natans*	Yes	LC	12S: DQ643833; 16S: DQ643833; AT6: DQ643833; COI: DQ643833; CYB: DQ643833	[S20]	
Faviidae	*Cyphastrea agassizi*	Yes	VU			Cyphastrea monophyly shown [S18]
Faviidae	*Cyphastrea chalcidicum*	Yes	LC	COI: AB117259; CYB: AB117336		
Faviidae	*Cyphastrea decadia*	Yes	LC			Cyphastrea monophyly shown [S18]; closest to C. japonica [S21]
Faviidae	*Cyphastrea hexasepta*	Yes	VU			Cyphastrea monophyly shown [S18]; closest to C. microphthalma [S9]
Faviidae	*Cyphastrea japonica*	Yes	LC			Cyphastrea monophyly shown [S18]
Faviidae	*Cyphastrea microphthalma*	Yes	LC	COI: FJ345416		
Faviidae	*Cyphastrea ocellina*	Yes	VU	12S: EF596996; 16S: L76132		
Faviidae	*Cyphastrea serailia*	Yes	LC	COI: AB117258; CYB: AB117334		
Faviidae	*Diploastrea heliopora*	Yes	NT	COI: AB117290; CYB: AB117375		
Faviidae	*Diploria clivosa*	Yes	LC	12S: EF597001; COI: AB117226; CYB: AB117304	[S20]	
Faviidae	*Diploria labyrinthiformis*	Yes	LC	12S: EF597002; COI: AB117224; CYB: AB117302	[S20]	

Family	Genus/Species	Reef	Red List	Molecular sources	Morphological sources	Remarks
Faviidae	*Diploria strigosa*	Yes	LC	12S: EF597003; COI: AB117225; CYB: AB117303	[S20]	
Faviidae	*Echinopora ashmorensis*	Yes	VU			Echinopora monophyly shown [S18]; closest to E. lamellosa [S7]
Faviidae	*Echinopora forskaliana*	Yes	NT			Echinopora monophyly shown [S18]
Faviidae	*Echinopora fruticulosa*	Yes	NT			Echinopora monophyly shown [S18]
Faviidae	*Echinopora gemmacea*	Yes	LC	COI: AB117263; CYB: AB117342		
Faviidae	*Echinopora grandicula*	Yes	DD			Echinopora monophyly shown [S18]
Faviidae	*Echinopora hirsutissima*	Yes	LC			Echinopora monophyly shown [S18]
Faviidae	*Echinopora horrida*	Yes	NT	COI: HQ203253		
Faviidae	*Echinopora irregularis*	Yes	DD			Echinopora monophyly shown [S18]; closest to E. hirsutissima [S9]
Faviidae	*Echinopora lamellosa*	Yes	LC	16S: L76003; COI: FJ345419		
Faviidae	*Echinopora mammiformis*	Yes	NT	COI: HQ203254		
Faviidae	*Echinopora pacificus*	Yes	NT	COI: AB117262; CYB: AB117341		
Faviidae	*Echinopora robusta*	Yes	VU			Echinopora monophyly shown [S18]; closest to E. forskaliana [S9]
Faviidae	*Echinopora taylorae*	Yes	NT			Echinopora monophyly shown [S18]
Faviidae	*Echinopora tiranensis*	Yes	DD			Echinopora monophyly shown [S18]
Faviidae	*Erythrastrea flabellata*	Yes	NT			Closest to Caulastraea [S22]
Faviidae	*Favia albidus*	Yes	NT			Clade VII-B monophyly shown [S18]; closest to F. matthaii [S9]
Faviidae	*Favia danae*	Yes	LC	COI: EU371663		
Faviidae	*Favia favus*	Yes	LC	12S: AF177048; COI: AB117267; CYB: AB117346		
Faviidae	*Favia fragum*	Yes	LC	12S: EF597005; 16S: U40295; COI: AB117222; CYB: AB117301	[S20]	

Family	Genus/Species	Reef	Red List	Molecular sources	Morphological sources	Remarks
Faviidae	*Favia gravida*	Yes	DD		[S20]	Previously excluded [S14]
Faviidae	*Favia helianthoides*	Yes	NT			Clade VII-B monophyly shown [S18]; closest to F. laxa [S8]
Faviidae	*Favia lacuna*	Yes	NT			Clade VII-B monophyly shown [S18]
Faviidae	*Favia laxa*	Yes	NT	COI: EU371707		Favia cf. laxa in GenBank
Faviidae	*Favia leptophylla*	Yes	DD	COI: AB117229; CYB: AB117307	[S5,S20]	
Faviidae	*Favia lizardensis*	Yes	NT	COI: HM018633		
Faviidae	*Favia maritima*	Yes	NT	COI: HQ203258		Favia cf. maritima in GenBank
Faviidae	*Favia marshae*	Yes	NT			Clade VII-F monophyly shown [S18]; closest to F. rotundata [S9]
Faviidae	*Favia matthaii*	Yes	NT	COI: HQ203259		
Faviidae	*Favia maxima*	Yes	NT	COI: HQ203260		
Faviidae	*Favia pallida*	Yes	LC	COI: AB117266; CYB: AB117345		
Faviidae	*Favia rosaria*	Yes	VU	COI: HQ203262		
Faviidae	*Favia rotumana*	Yes	LC	COI: FJ345427		
Faviidae	*Favia rotundata*	Yes	NT	COI: HQ203263		Clade VII-F monophyly shown [S18]
Faviidae	*Favia speciosa*	Yes	LC	COI: AB441194; CYB: AB441279		
Faviidae	*Favia stelligera*	Yes	NT	COI: AB117264; CYB: AB117343		
Faviidae	*Favia truncatus*	Yes	LC	COI: HQ203266		
Faviidae	*Favia veroni*	Yes	NT			Clade VII-B monophyly shown [S18]; closest to F. maxima [S21]
Faviidae	*Favia vietnamensis*	Yes	NT			Clade VII-B monophyly shown [S18]
Faviidae	*Favites abdita*	Yes	NT	COI: HQ203267	[S5]	
Faviidae	*Favites acuticollis*	Yes	NT			Clade VII-F monophyly shown [S18]
Faviidae	*Favites bestae*	Yes	NT			Clade VII-F monophyly shown [S18]
Faviidae	*Favites chinensis*	Yes	NT	COI: AB117269; CYB: AB117349		
Faviidae	*Favites complanata*	Yes	NT	COI: EU371689		
Faviidae	*Favites flexuosa*	Yes	NT	COI: HQ203269		

**Family**	**Genus/Species**	**Reef**	**Red List**	**Molecular sources**	**Morphological sources**	**Remarks**
Faviidae	*Favites halicora*	Yes	NT	COI: AB117268; CYB: AB117348		
Faviidae	*Favites micropentagona*	Yes	NT			Closest to F. pentagona [S9]
Faviidae	*Favites paraflexuosa*	Yes	NT	COI: EU371694		
Faviidae	*Favites pentagona*	Yes	LC	COI: HQ203271		
Faviidae	*Favites russelli*	Yes	NT	COI: HQ203272		
Faviidae	*Favites spinosa*	Yes	VU			Clade VII-F monophyly shown [S18]
Faviidae	*Favites stylifera*	Yes	NT	COI: HQ203273	[S10]	Clade VII-G monophyly shown [S18]
Faviidae	*Favites vasta*	Yes	NT			Clade VII-F monophyly shown [S18]
Faviidae	*Goniastrea aspera*	Yes	LC	COI: AB117271; CYB: AB117351		
Faviidae	*Goniastrea australensis*	Yes	LC	COI: HQ203274		
Faviidae	*Goniastrea columella*	Yes	NT			Closest to G. pectinata [S8]
Faviidae	*Goniastrea deformis*	Yes	VU	COI: AB441195; CYB: AB441280		
Faviidae	*Goniastrea edwardsi*	Yes	LC	COI: EU371697		
Faviidae	*Goniastrea favulus*	Yes	NT	COI: EU371698		
Faviidae	*Goniastrea minuta*	Yes	NT			Closest to G. retiformis [S9]
Faviidae	*Goniastrea palauensis*	Yes	NT	COI: EU371699		Clade VII-B monophyly shown [S18]
Faviidae	*Goniastrea pectinata*	Yes	LC	COI: AB117270; CYB: AB117350		
Faviidae	*Goniastrea peresi*	Yes	NT			Closest to G. aspera [S8]
Faviidae	*Goniastrea ramosa*	Yes	VU			Closest to G. retiformis [S9]
Faviidae	*Goniastrea retiformis*	Yes	LC	12S: EF597033; COI: HQ203275		
Faviidae	*Goniastrea thecata*	Yes	NT			Closest to G. aspera [S9]
Faviidae	*Leptastrea aequalis*	Yes	VU			Leptastrea + Fungiidae monophyly shown [S23]
Faviidae	*Leptastrea bewickensis*	Yes	NT			Leptastrea + Fungiidae monophyly shown [S23]
Faviidae	*Leptastrea bottae*	Yes	NT			Leptastrea + Fungiidae monophyly shown [S23]
Faviidae	*Leptastrea inaequalis*	Yes	NT			Leptastrea + Fungiidae monophyly shown [S23]
Faviidae	*Leptastrea pruinosa*	Yes	LC	COI: AB441196; CYB: AB441281		
Faviidae	*Leptastrea purpurea*	Yes	LC	COI: EU371702		

Family	Genus/Species	Reef	Red List	Molecular sources	Morphological sources	Remarks
Faviidae	*Leptastrea transversa*	Yes	LC	COI: HM018655		
Faviidae	*Leptoria irregularis*	Yes	VU	COI: AB117272; CYB: AB117353		
Faviidae	*Leptoria phrygia*	Yes	NT	16S: L76011; COI: AB117273; CYB: AB117354		
Faviidae	*Manicina areolata*	Yes	LC	12S: EF597012; COI: AB117227; CYB: AB117305	[S20]	
Faviidae	*Montastraea annularis*	Yes	EN	12S: AP008973; 16S: AP008973; AT6: AP008973; COI: AP008973; CYB: AP008973		
Faviidae	*Montastraea annuligera*	Yes	NT	COI: JN248781		Clade VII-B monophyly shown [S18]
Faviidae	*Montastraea cavernosa*	Yes	LC	12S: EF597006; COI: AB117288; CYB: AB117373	[S5]	
Faviidae	*Montastraea colemani*	Yes	NT	COI: HQ203284		Clade VII-F monophyly shown [S18]
Faviidae	*Montastraea curta*	Yes	LC	COI: AB117278; CYB: AB117359		
Faviidae	*Montastraea faveolata*	Yes	EN	12S: AP008977; 16S: AP008977; AT6: AP008977; COI: AP008977; CYB: AP008977		
Faviidae	*Montastraea franksi*	Yes	VU	12S: AP008976; 16S: AP008976; AT6: AP008976; COI: AP008976; CYB: AP008976		
Faviidae	*Montastraea magnistellata*	Yes	NT	COI: AB117279; CYB: AB117360		Clade VII-F monophyly shown [S18]
Faviidae	*Montastraea multipunctata*	Yes	VU	COI: HQ203289		
Faviidae	*Montastraea salebrosa*	Yes	VU	COI: HQ203290		

Family	Genus/Species	Reef	Red List	Molecular sources	Morphological sources	Remarks
Faviidae	*Montastraea serageldini*	Yes	VU			Closest to M. curta [S9]
Faviidae	*Montastraea valenciennesi*	Yes	NT	12S: AF333061; COI: AB117280; CYB: AB117361		Clade VII-F monophyly shown [S18]
Faviidae	*Moseleya latistellata*	Yes	VU	COI: HQ203293		
Faviidae	*Oulastrea crispata*	Yes	LC	12S: AF333062; COI: AB441197; CYB: AB441282		
Faviidae	*Oulophyllia bennettae*	Yes	NT	COI: AB117277; CYB: AB117358		
Faviidae	*Oulophyllia crispa*	Yes	NT	COI: AB117276; CYB: AB117357		
Faviidae	*Oulophyllia levis*	Yes	LC			Closest to O. crispa [S8]
Faviidae	*Parasimplastrea sheppardi*	Yes	EN			Closest to Leptastrea [S9]
Faviidae	*Platygyra acuta*	Yes	NT	COI: JN248782	[S10]	
Faviidae	*Platygyra carnosus*	Yes	NT		[S10]	
Faviidae	*Platygyra contorta*	Yes	LC	COI: JN248783	[S10]	
Faviidae	*Platygyra crosslandi*	Yes	NT		[S10]	
Faviidae	*Platygyra daedalea*	Yes	LC	COI: AB117281; CYB: AB117362	[S5,S10]	
Faviidae	*Platygyra lamellina*	Yes	NT	COI: HQ203302; CYB: AB117363	[S10]	
Faviidae	*Platygyra pini*	Yes	LC	COI: HQ203303	[S10]	
Faviidae	*Platygyra ryukyuensis*	Yes	NT	COI: HQ203304	[S10]	
Faviidae	*Platygyra sinensis*	Yes	LC	12S: AF177047; COI: HQ203305	[S10]	
Faviidae	*Platygyra verweyi*	Yes	NT	COI: EU371722	[S10]	Platygyra cf. verweyi in GenBank
Faviidae	*Platygyra yaeyamaensis*	Yes	VU		[S10]	
Faviidae	*Plesiastrea devantieri*	Yes	NT	COI: FR837987		
Faviidae	*Plesiastrea versipora*	Yes	LC	COI: AB289561; CYB: AB289566		
Faviidae	*Solenastrea bournoni*	Yes	LC	COI: AB117291; CYB: AB117376		
Faviidae	*Solenastrea hyades*	Yes	LC	COI: FJ966870		

Family	Genus/Species	Reef	Red List	Molecular sources	Morphological sources	Remarks
Flabellidae	*Flabellum angulare*	No	N/A	16S: AF550363	[S10]	
Flabellidae	*Flabellum apertum*	No	N/A	COI: HM018635	[S10]	
Flabellidae	*Flabellum arcuatile*	No	N/A	COI: HM018636	[S10]	
Flabellidae	*Flabellum deludens*	No	N/A	16S: AB510170; COI: HM018638	[S10]	
Flabellidae	*Flabellum folkesoni*	No	N/A	COI: HM018639	[S10]	
Flabellidae	*Flabellum impensum*	No	N/A	16S: AF265582	[S10]	
Flabellidae	*Flabellum japonicum*	No	N/A	16S: AB510178	[S10]	
Flabellidae	*Flabellum lamellulosum*	No	N/A	COI: HM018640	[S10]	
Flabellidae	*Flabellum lowekeyesi*	No	N/A	COI: HM018641	[S10]	
Flabellidae	*Flabellum magnificum*	No	N/A	16S: AB510167; COI: HM018637	[S10]	Flabellum cf. magnificum in GenBank
Flabellidae	*Flabellum pavoninum*	No	N/A	16S: AB510168	[S10]	
Flabellidae	*Flabellum tuthilli*	No	N/A	COI: HM018643	[S10]	
Flabellidae	*Flabellum vaughani*	No	N/A	COI: HM018644	[S10]	
Flabellidae	*Javania exserta*	No	N/A	COI: HM018651		
Flabellidae	*Javania fusca*	No	N/A	COI: HM018652		
Flabellidae	*Javania insignis*	No	N/A	16S: AB510174		
Flabellidae	*Javania lamprotichum*	No	N/A	COI: HM018653		
Flabellidae	*Monomyces pygmaea*	No	N/A	16S: AF265583	[S10]	
Flabellidae	*Placotrochides scaphula*	No	N/A	COI: HM018661		
Flabellidae	*Placotrochus laevis*	No	N/A	16S: AF265589	[S10]	
Flabellidae	*Rhizotrochus typus*	No	N/A	16S: AB510175		
Flabellidae	*Truncatoflabellum australiensis*	No	N/A	COI: HM018670		
Flabellidae	*Truncatoflabellum candeanum*	No	N/A	COI: HM018671		
Flabellidae	*Truncatoflabellum macroeschara*	No	N/A	COI: HM018672		
Flabellidae	*Truncatoflabellum spheniscus*	No	N/A	16S: AB510172		
Fungiacyathidae	*Fungiacyathus fragilis*	No	N/A	COI: HM018645		
Fungiacyathidae	*Fungiacyathus marenzelleri*	No	N/A	12S: EF597074; 16S: L76004		
Fungiacyathidae	*Fungiacyathus pusillus*	No	N/A	COI: HM018646		

Family	Genus/Species	Reef	Red List	Molecular sources	Morphological sources	Remarks
Fungiacyathidae	*Fungiacyathus stephanus*	No	N/A	12S: JF825138; 16S: JF825138; AT6: JF825138; COI: JF825138; CYB: JF825138; ND5: JF825138		
Fungiacyathidae	*Fungiacyathus turbinolioides*	No	N/A	COI: HM018648		
Fungiidae	*Cantharellus doederleini*	Yes	LC		[S24]	
Fungiidae	*Cantharellus jebbi*	Yes	LC			Cantharellus monophyly assumed
Fungiidae	*Cantharellus noumeae*	Yes	EN		[S24]	
Fungiidae	*Ctenactis albitentaculata*	Yes	NT	COI: EU149869	[S24]	
Fungiidae	*Ctenactis crassa*	Yes	LC	COI: EU149859	[S24]	
Fungiidae	*Ctenactis echinata*	Yes	LC	COI: EU149879	[S24]	
Fungiidae	*Cycloseris costulata*	Yes	LC	COI: EU149870	[S5,S24]	Revised from Fungia costulata & C. marginata
Fungiidae	*Cycloseris curvata*	Yes	VU		[S24]	Revised from Fungia curvata
Fungiidae	*Cycloseris cyclolites*	Yes	LC	COI: EU202719	[S24]	Revised from Fungia cyclolites
Fungiidae	*Cycloseris distorta*	Yes	LC		[S24]	Revised from Fungia distorta
Fungiidae	*Cycloseris fragilis*	Yes	LC	16S: L75998; COI: EU149860	[S24]	Revised from Fungia fragilis
Fungiidae	*Cycloseris hexagonalis*	Yes	LC		[S24]	Revised from Fungia hexagonalis
Fungiidae	*Cycloseris mokai*	Yes	LC	COI: EU149877	[S5,S24]	Revised from Lithophyllon mokai
Fungiidae	*Cycloseris sinensis*	Yes	LC	COI: EU149900	[S5,S24]	Revised from Fungia sinensis
Fungiidae	*Cycloseris somervillei*	Yes	LC		[S24]	Revised from Fungia somervillei
Fungiidae	*Cycloseris tenuis*	Yes	LC	COI: EU149871	[S24]	Revised from Fungia tenuis
Fungiidae	*Cycloseris vaughani*	Yes	LC	16S: L75999; COI: EU149861	[S24]	Revised from Fungia vaughani
Fungiidae	*Danafungia horrida*	Yes	LC		[S24]	Revised from Fungia horrida
Fungiidae	*Danafungia scruposa*	Yes	LC	COI: EU149872	[S24]	Revised from Fungia scruposa
Fungiidae	*Fungia fungites*	Yes	NT	COI: EU149892	[S5,S10,S24]	
Fungiidae	*Halomitra clavator*	Yes	VU	COI: EU149904	[S24]	
Fungiidae	*Halomitra pileus*	Yes	LC	COI: EU149865	[S5,S24]	
Fungiidae	*Heliofungia actiniformis*	Yes	VU	12S: EF596995; COI: EU149885	[S24]	

Family	Genus/Species	Reef	Red List	Molecular sources	Morphological sources	Remarks
Fungiidae	*Heliofungia fralinae*	Yes	LC	COI: EU149901	[S24]	Revised from Fungia fralinae
Fungiidae	*Herpolitha limax*	Yes	LC	COI: AB441223; CYB: AB441308	[S5,S24]	
Fungiidae	*Lithophyllon concinna*	Yes	LC	COI: EU149893	[S5,S24]	Revised from Fungia concinna
Fungiidae	*Lithophyllon puishani*	Yes	DD			Revised from Fungia puishani; closest to L. scabra [S9]
Fungiidae	*Lithophyllon ranjithi*	Yes	EN			Lithophyllon monophyly shown [S25]
Fungiidae	*Lithophyllon repanda*	Yes	LC	COI: EU149883	[S5,S24]	Revised from Fungia repanda
Fungiidae	*Lithophyllon scabra*	Yes	LC	COI: EU149874	[S24]	Revised from Fungia scabra
Fungiidae	*Lithophyllon spinifer*	Yes	LC	COI: EU149864	[S24]	Revised from Fungia spinifer
Fungiidae	*Lithophyllon undulatum*	Yes	NT	COI: EU149867	[S24]	
Fungiidae	*Lobactis scutaria*	Yes	LC	12S: DQ320497; 16S: L76005; COI: AB441224; CYB: AB441309	[S24]	Revised from Fungia scutaria
Fungiidae	*Pleuractis granulosa*	Yes	LC	COI: EU149884	[S24,S26]	Revised from Fungia granulosa
Fungiidae	*Pleuractis gravis*	Yes	DD	COI: EU149910	[S24,S26]	Previously excluded [S14]; revised from Fungia gravis
Fungiidae	*Pleuractis moluccensis*	Yes	LC	COI: EU149909	[S24,S26]	Revised from Fungia moluccensis
Fungiidae	*Pleuractis paumotensis*	Yes	LC	COI: EU149911	[S24,S26]	Revised from Fungia paumotensis
Fungiidae	*Pleuractis seychellensis*	Yes	VU		[S26]	Revised from Fungia seychellensis
Fungiidae	*Pleuractis taiwanensis*	Yes	VU		[S26]	Revised from Fungia taiwanensis
Fungiidae	*Podabacia crustacea*	Yes	LC	COI: EU149878	[S24]	
Fungiidae	*Podabacia kunzmanni*	Yes	DD	COI: EU149908		New species [S27]
Fungiidae	*Podabacia motuporensis*	Yes	NT	COI: EU149868		
Fungiidae	*Podabacia sinai*	Yes	DD	COI: EU149888		
Fungiidae	*Polyphyllia novaehiberniae*	Yes	NT		[S24]	
Fungiidae	*Polyphyllia talpina*	Yes	LC	COI: EU149915	[S5,S24]	
Fungiidae	*Sandalolitha dentata*	Yes	LC	COI: EU149914	[S24]	
Fungiidae	*Sandalolitha robusta*	Yes	LC	COI: EU149917	[S24]	
Fungiidae	*Zoopilus echinatus*	Yes	LC	12S: EF596990; 16S: L76024; COI: EU149916	[S5,S10,S24]	

Family	Genus/Species	Reef	Red List	Molecular sources	Morphological sources	Remarks
Gardineriidae	*Gardineria hawaiiensis*	No	N/A	12S: GQ868660; 16S: GQ868701; COI: GQ868678		
Gardineriidae	*Gardineria paradoxa*	No	N/A	12S: GQ868656; 16S: GQ868700; COI: GQ868681		
Guyniidae	*Guynia annulata*	No	N/A	16S: AF265580		
Meandrinidae	*Ctenella chagius*	Yes	EN	COI: AB441208; CYB: AB441293		
Meandrinidae	*Dendrogyra cylindrus*	Yes	VU	12S: EF597024; COI: AB117299; CYB: AB117384		
Meandrinidae	*Dichocoenia stellaris*	Yes	DD		[S10]	
Meandrinidae	*Dichocoenia stokesi*	Yes	VU	12S: EF597020; 16S: AF265607; COI: AB117298; CYB: AB117383	[S10]	
Meandrinidae	*Eusmilia fastigiata*	Yes	LC	COI: AB117294; CYB: AB117380		
Meandrinidae	*Gyrosmilia interrupta*	Yes	LC			Closest to Ctenella chagius [S8]
Meandrinidae	*Meandrina braziliensis*	Yes	DD	COI: AB117297; CYB: AB117382	[S5]	
Meandrinidae	*Meandrina danae*	Yes	LC			Closest to M. braziliensis [S28]
Meandrinidae	*Meandrina jacksoni*	Yes	N/A			New species, closest to M. meandrites [S29]
Meandrinidae	*Meandrina meandrites*	Yes	LC	12S: EF597032; COI: AB117296; CYB: AB117381		
Meandrinidae	*Montigyra kenti*	Yes	DD			Closest to Gyrosmilia [S6]
Merulinidae	*Boninastrea boninensis*	Yes	DD			Closest to Merulina [S6]
Merulinidae	*Hydnophora bonsai*	Yes	EN		[S10]	
Merulinidae	*Hydnophora exesa*	Yes	NT	12S: AF333059; COI: AB117285; CYB: AB117370	[S10]	

Family	Genus/Species	Reef	Red List	Molecular sources	Morphological sources	Remarks
Merulinidae	*Hydnophora grandis*	Yes	LC	COI: AB117286; CYB: AB117371	[S10]	
Merulinidae	*Hydnophora microconos*	Yes	NT	COI: HQ203277	[S10]	
Merulinidae	*Hydnophora pilosa*	Yes	LC	COI: HQ203278	[S10]	
Merulinidae	*Hydnophora rigida*	Yes	LC	12S: EF597000; 16S: L76009	[S10]	
Merulinidae	*Merulina ampliata*	Yes	LC	12S: AF333058; COI: AB117283; CYB: AB117368		
Merulinidae	*Merulina scabricula*	Yes	LC	16S: L76014; COI: AB117284; CYB: AB117369		
Merulinidae	*Merulina scheeri*	Yes	LC			Merulina monophyly shown [S18]; closest to M. ampliata [S30]
Merulinidae	*Paraclavarina triangularis*	Yes	NT			Closest to Merulina [S31]
Merulinidae	*Scapophyllia cylindrica*	Yes	LC	COI: AB441198; CYB: AB441283		
Micrabaciidae	*Leptopenus antarcticus*	No	N/A		[S5]	
Micrabaciidae	*Letepsammia formosissima*	No	N/A	12S: GQ868663; 16S: GQ868697; COI: GQ868685		
Micrabaciidae	*Rhombopsammia niphada*	No	N/A	12S: GQ868661; 16S: GQ868693; COI: GQ868683		
Micrabaciidae	*Stephanophyllia complicata*	No	N/A	16S: GQ868689		
Mussidae	*Acanthastrea bowerbanki*	Yes	VU		[S32]	
Mussidae	*Acanthastrea brevis*	Yes	VU		[S32]	
Mussidae	*Acanthastrea echinata*	Yes	LC	COI: AB117249; CYB: AB117327	[S20,S32]	
Mussidae	*Acanthastrea faviaformis*	Yes	VU		[S32]	
Mussidae	*Acanthastrea hemprichii*	Yes	VU		[S32]	
Mussidae	*Acanthastrea hillae*	Yes	NT	COI: AB441199; CYB: AB441284		
Mussidae	*Acanthastrea ishigakiensis*	Yes	VU		[S32]	

Family	Genus/Species	Reef	Red List	Molecular sources	Morphological sources	Remarks
Mussidae	*Acanthastrea lordhowensis*	Yes	NT		[S32]	
Mussidae	*Acanthastrea maxima*	Yes	NT		[S32]	
Mussidae	*Acanthastrea regularis*	Yes	VU		[S32]	
Mussidae	*Acanthastrea rotundoflora*	Yes	NT	COI: AB117251; CYB: AB117328	[S20,S32]	
Mussidae	*Acanthastrea subechinata*	Yes	NT		[S32]	
Mussidae	*Australomussa rowleyensis*	Yes	NT		[S32]	
Mussidae	*Blastomussa merleti*	Yes	LC		[S32]	
Mussidae	*Blastomussa wellsi*	Yes	NT	COI: AB289563; CYB: AB289565	[S32]	
Mussidae	*Cynarina lacrymalis*	Yes	NT	12S: EF597034; COI: AB117246; CYB: AB117323	[S32]	
Mussidae	*Indophyllia macassarensis*	Yes	DD			Closest to Cynarina lacrymalis [S33]
Mussidae	*Isophyllastrea rigida*	Yes	LC		[S20,S32]	
Mussidae	*Isophyllia sinuosa*	Yes	LC	COI: AB117238; CYB: AB117315	[S20,S32]	
Mussidae	*Lobophyllia corymbosa*	Yes	LC	COI: AB117241; CYB: AB117318	[S10,S32]	
Mussidae	*Lobophyllia dentatus*	Yes	VU		[S10,S32]	
Mussidae	*Lobophyllia diminuta*	Yes	VU		[S10,S32]	
Mussidae	*Lobophyllia flabelliformis*	Yes	VU		[S10,S32]	
Mussidae	*Lobophyllia hataii*	Yes	LC		[S10,S32]	
Mussidae	*Lobophyllia hemprichii*	Yes	LC	12S: EF597013; 16S: L76013; COI: AB117240; CYB: AB117317	[S5,S10,S32]	
Mussidae	*Lobophyllia pachysepta*	Yes	NT	COI: AB117242; CYB: AB117319	[S10,S32]	
Mussidae	*Lobophyllia robusta*	Yes	LC		[S10,S32]	
Mussidae	*Lobophyllia serratus*	Yes	EN		[S10,S32]	
Mussidae	*Micromussa amakusensis*	Yes	NT	COI: AB441200; CYB: AB441285		

Family	Genus/Species	Reef	Red List	Molecular sources	Morphological sources	Remarks
Mussidae	*Micromussa diminuta*	Yes	DD			Micromussa monophyly assumed; closest to M. minuta [S9]
Mussidae	*Micromussa minuta*	Yes	NT			Micromussa monophyly assumed
Mussidae	*Mussa angulosa*	Yes	LC	12S: DQ643834; 16S: DQ643834; AT6: DQ643834; COI: DQ643834; CYB: DQ643834	[S20,S32]	
Mussidae	*Mussismilia braziliensis*	Yes	DD	COI: AB117231; CYB: AB117309	[S5,S20,S32]	
Mussidae	*Mussismilia harttii*	Yes	DD	COI: AB117232; CYB: AB117308	[S5,S20,S32]	
Mussidae	*Mussismilia hispida*	Yes	DD	COI: AB117233; CYB: AB117310	[S5,S20,S32]	
Mussidae	*Mycetophyllia aliciae*	Yes	LC	12S: EF597039; COI: AB117235; CYB: AB117312	[S20,S32]	
Mussidae	*Mycetophyllia danaana*	Yes	LC	COI: AB117234; CYB: AB117311	[S20,S32]	
Mussidae	*Mycetophyllia ferox*	Yes	VU		[S20,S32]	
Mussidae	*Mycetophyllia lamarckiana*	Yes	LC	12S: EF597040	[S20,S32]	
Mussidae	*Mycetophyllia reesi*	Yes	DD		[S20,S32]	
Mussidae	*Scolymia australis*	Yes	LC		[S32]	
Mussidae	*Scolymia cubensis*	Yes	LC	COI: AB117236; CYB: AB117314	[S20]	Atlantic Scolymia
Mussidae	*Scolymia lacera*	Yes	LC		[S20]	Junior synonym of S. cubensis [S8]; Atlantic Scolymia
Mussidae	*Scolymia vitiensis*	Yes	NT	COI: AB117247; CYB: AB117324	[S32]	
Mussidae	*Scolymia wellsi*	Yes	DD		[S5,S20]	Junior synonym of S. cubensis [S34]; Atlantic Scolymia
Mussidae	*Symphyllia agaricia*	Yes	LC	COI: AB117243; CYB: AB117320	[S32]	
Mussidae	*Symphyllia erythraea*	Yes	LC		[S32]	

Family	Genus/Species	Reef	Red List	Molecular sources	Morphological sources	Remarks
Mussidae	*Symphyllia hassi*	Yes	VU		[S32]	
Mussidae	*Symphyllia radians*	Yes	LC	COI: AB117245; CYB: AB117322	[S32]	
Mussidae	*Symphyllia recta*	Yes	LC	COI: AB117244; CYB: AB117321	[S32]	
Mussidae	*Symphyllia valenciennesii*	Yes	LC	COI: HM018666	[S32]	
Mussidae	*Symphyllia wilsoni*	Yes	LC		[S32]	
Oculinidae	*Cyathelia axillaris*	No	N/A	COI: HM018622		
Oculinidae	*Galaxea acrhelia*	Yes	VU			Closest to G. horrescens [S9]
Oculinidae	*Galaxea astreata*	Yes	VU	12S: AF333056		
Oculinidae	*Galaxea cryptoramosa*	Yes	VU			Closest to G. astreata [S9]
Oculinidae	*Galaxea fascicularis*	Yes	NT	16S: L76006; COI: AB441201; CYB: AB441286; ND5: AB109376		
Oculinidae	*Galaxea horrescens*	Yes	LC	12S: EF597096; 16S: L75994		
Oculinidae	*Galaxea longisepta*	Yes	NT			Closest to G. horrescens [S9]
Oculinidae	*Galaxea paucisepta*	Yes	NT			Closest to G. astreata [S35]
Oculinidae	*Madrepora oculata*	No	N/A	16S: AF550369; COI: HM018659		
Oculinidae	*Oculina diffusa*	Yes	LC	COI: AB117293; CYB: AB117379	[S10]	
Oculinidae	*Oculina patagonica*	Yes	LC	12S: EF597025; 16S: AF265601	[S5,S10]	
Oculinidae	*Oculina robusta*	Yes	DD	COI: FJ966869	[S10]	
Oculinidae	*Oculina tenella*	Yes	DD		[S10]	
Oculinidae	*Oculina valenciennesi*	Yes	DD		[S10]	
Oculinidae	*Oculina varicosa*	Yes	VU	COI: FJ966875	[S10]	
Oculinidae	*Schizoculina africana*	Yes	DD			Schizoculina monophyly assumed; closest to Oculina [S36]
Oculinidae	*Schizoculina fissipara*	Yes	DD			Schizoculina monophyly assumed; closest to Oculina [S36]
Oculinidae	*Simplastrea vesicularis*	Yes	DD			Closest to Galaxea [S8]

Family	Genus/Species	Reef	Red List	Molecular sources	Morphological sources	Remarks
Pectiniidae	*Echinomorpha nishihirai*	Yes	NT			Closest to Echinophyllia [S7]
Pectiniidae	*Echinophyllia aspera*	Yes	LC	COI: AB117252; CYB: AB117329		
Pectiniidae	*Echinophyllia costata*	Yes	VU			Echinophyllia monophyly shown [S37]; closest to E. pectinata [S9]
Pectiniidae	*Echinophyllia echinata*	Yes	LC			Echinophyllia monophyly shown [S37]
Pectiniidae	*Echinophyllia echinoporoides*	Yes	LC	COI: AB117254; CYB: AB117331		
Pectiniidae	*Echinophyllia orpheensis*	Yes	LC	12S: AF333065; COI: AB117253; CYB: AB117330		
Pectiniidae	*Echinophyllia patula*	Yes	LC			Echinophyllia monophyly shown [S37]; closest to E. aspera [S8]
Pectiniidae	*Echinophyllia pectinata*	Yes	DD			Echinophyllia monophyly shown [S37]
Pectiniidae	*Mycedium elephantotus*	Yes	LC	12S: AF333057; COI: AB117387; CYB: AB117366		
Pectiniidae	*Mycedium mancaoi*	Yes	LC			Mycedium monophyly shown [S18]; closest to M. elephantotus [S8]
Pectiniidae	*Mycedium robokaki*	Yes	LC	COI: HQ203295		
Pectiniidae	*Mycedium spina*	Yes	DD			Mycedium monophyly shown [S18]
Pectiniidae	*Mycedium steeni*	Yes	VU			Mycedium monophyly shown [S18]; closest to M. robokaki [S9]
Pectiniidae	*Mycedium umbra*	Yes	LC			Mycedium monophyly shown [S18]; closest to M. elephantotus [S9]
Pectiniidae	*Oxypora convoluta*	Yes	DD			Oxypora monophyly assumed; closest to O. lacera [S9]
Pectiniidae	*Oxypora crassispinosa*	Yes	LC			Oxypora monophyly assumed; closest to O. glabra [S8]
Pectiniidae	*Oxypora egyptensis*	Yes	DD			Oxypora monophyly assumed; closest to O. glabra [S8]
Pectiniidae	*Oxypora glabra*	Yes	LC			Oxypora monophyly assumed
Pectiniidae	*Oxypora lacera*	Yes	LC	COI: AB117255; CYB: AB117332		

Family	Genus/Species	Reef	Red List	Molecular sources	Morphological sources	Remarks
Pectiniidae	*Pectinia africanus*	Yes	VU			Pectinia + Mycedium monophyly shown [S18]; closest to P. lactuca [S9]
Pectiniidae	*Pectinia alcicornis*	Yes	VU	12S: EF597037; 16S: L76017; COI: AB117385; CYB: AB117364		
Pectiniidae	*Pectinia ayleni*	Yes	NT	COI: HQ203299		
Pectiniidae	*Pectinia crassa*	Yes	DD			Pectinia + Mycedium monophyly shown [S18]
Pectiniidae	*Pectinia elongata*	Yes	NT			Pectinia + Mycedium monophyly shown [S18]
Pectiniidae	*Pectinia lactuca*	Yes	VU	COI: HQ203300		
Pectiniidae	*Pectinia maxima*	Yes	EN			Pectinia + Mycedium monophyly shown [S18]; closest to P. lactuca & P. ayleni [S8]
Pectiniidae	*Pectinia paeonia*	Yes	NT	COI: AB117386; CYB: AB117365	[S5]	
Pectiniidae	*Pectinia pygmaeus*	Yes	NT			Pectinia + Mycedium monophyly shown [S18]; closest to P. elongata & P. teres [S9]
Pectiniidae	*Pectinia teres*	Yes	NT			Pectinia + Mycedium monophyly shown [S18]; closest to P. elongata [S8]
Pocilloporidae	*Pocillopora ankeli*	Yes	VU		[S1]	
Pocilloporidae	*Pocillopora capitata*	Yes	LC		[S1]	
Pocilloporidae	*Pocillopora damicornis*	Yes	LC	12S: EF526302; 16S: EF526302; AT6: EF526302; COI: EF526302; CYB: EF526302	[S1]	
Pocilloporidae	*Pocillopora danae*	Yes	VU		[S1]	
Pocilloporidae	*Pocillopora effusus*	Yes	DD		[S1]	
Pocilloporidae	*Pocillopora elegans*	Yes	VU		[S1]	
Pocilloporidae	*Pocillopora eydouxi*	Yes	NT	12S: EF526303; 16S: EF526303; AT6: EF526303; COI: EF526303; CYB: EF526303	[S1]	
Pocilloporidae	*Pocillopora fungiformis*	Yes	EN		[S1]	

Family	Genus/Species	Reef	Red List	Molecular sources	Morphological sources	Remarks
Pocilloporidae	*Pocillopora indiania*	Yes	VU		[S1]	
Pocilloporidae	*Pocillopora inflata*	Yes	VU		[S1]	
Pocilloporidae	*Pocillopora kelleheri*	Yes	LC		[S1]	
Pocilloporidae	*Pocillopora ligulata*	Yes	LC		[S1]	
Pocilloporidae	*Pocillopora meandrina*	Yes	LC	12S: EF596976; 16S: L76018	[S1]	
Pocilloporidae	*Pocillopora molokensis*	Yes	DD		[S1]	
Pocilloporidae	*Pocillopora setichelli*	Yes	LC		[S1]	
Pocilloporidae	*Pocillopora verrucosa*	Yes	LC	COI: AB441230; CYB: AB441315	[S1]	
Pocilloporidae	*Pocillopora woodjonesi*	Yes	LC		[S1]	
Pocilloporidae	*Pocillopora zelli*	Yes	LC		[S1]	
Pocilloporidae	*Seriatopora aculeata*	Yes	VU			Seriatopora monophyly shown [S37]; closest to S. stellata [S8]
Pocilloporidae	*Seriatopora caliendrum*	Yes	NT	12S: EF633601; 16S: EF633601; AT6: EF633601; COI: EF633601; CYB: EF633601		
Pocilloporidae	*Seriatopora dendritica*	Yes	VU			Seriatopora monophyly shown [S37]; closest to S. hystrix [S9]
Pocilloporidae	*Seriatopora guttatus*	Yes	LC			Seriatopora monophyly shown [S37]; closest to S. hystrix [S9]
Pocilloporidae	*Seriatopora hystrix*	Yes	LC	12S: EF633600; 16S: EF633600; AT6: EF633600; COI: EF633600; CYB: EF633600		
Pocilloporidae	*Seriatopora stellata*	Yes	NT			Seriatopora monophyly shown [S37]
Pocilloporidae	*Stylophora danae*	Yes	LC			Stylophora monophyly assumed; closest to S. pistillata [S8]
Pocilloporidae	*Stylophora kuehlmanni*	Yes	LC			Stylophora monophyly assumed; closest to S. subseriata [S8]

Family	Genus/Species	Reef	Red List	Molecular sources	Morphological sources	Remarks
Pocilloporidae	*Stylophora madagascarensis*	Yes	EN			Stylophora monophyly assumed; closest to S. kuehlmanni & S. subseriata [S9]
Pocilloporidae	*Stylophora mamillata*	Yes	LC			Stylophora monophyly assumed; not close to other Stylophora [S8]
Pocilloporidae	*Stylophora pistillata*	Yes	NT	12S: EU400214; 16S: EU400214; AT6: EU400214; COI: EU400214; CYB: EU400214		
Pocilloporidae	*Stylophora subseriata*	Yes	LC			Stylophora monophyly assumed
Pocilloporidae	*Stylophora wellsi*	Yes	NT			Stylophora monophyly assumed
Poritidae	*Alveopora allingi*	Yes	VU			Alveopora monophyly assumed
Poritidae	*Alveopora catalai*	Yes	NT			Alveopora monophyly assumed; closest to A. allingi & A. gigas [S8]
Poritidae	*Alveopora daedalea*	Yes	VU	12S: EF597088; 16S: AF265592; COI: AB441245; CYB: AB441330		Alveopora sp. in GenBank
Poritidae	*Alveopora excelsa*	Yes	EN			Alveopora monophyly assumed
Poritidae	*Alveopora fenestrata*	Yes	VU			Alveopora monophyly assumed; closest to A. marionensis & A. verrilliana [S8]
Poritidae	*Alveopora gigas*	Yes	VU			Alveopora monophyly assumed; closest to A. allingi & A. catalai [S8]
Poritidae	*Alveopora japonica*	Yes	VU			Alveopora monophyly assumed; closest to A. tizardi [S8]
Poritidae	*Alveopora marionensis*	Yes	VU			Alveopora monophyly assumed; closest to A. fenestrata & A. verrilliana [S8]
Poritidae	*Alveopora minuta*	Yes	EN			Alveopora monophyly assumed; closest to A. viridis [S9]
Poritidae	*Alveopora ocellata*	Yes	DD			Alveopora monophyly assumed
Poritidae	*Alveopora spongiosa*	Yes	NT			Alveopora monophyly assumed; closest to A. daedalea [S8]
Poritidae	*Alveopora tizardi*	Yes	LC			Alveopora monophyly assumed
Poritidae	*Alveopora verrilliana*	Yes	VU			Alveopora monophyly assumed

Family	Genus/Species	Reef	Red List	Molecular sources	Morphological sources	Remarks
Poritidae	*Alveopora viridis*	Yes	NT			Alveopora monophyly assumed
Poritidae	*Goniopora albiconus*	Yes	VU		[S10]	
Poritidae	*Goniopora burgosi*	Yes	VU		[S10]	
Poritidae	*Goniopora cellulosa*	Yes	VU		[S10]	
Poritidae	*Goniopora ciliatus*	Yes	LC		[S10]	
Poritidae	*Goniopora columna*	Yes	NT	12S: JF825141; 16S: JF825141; AT6: JF825141; COI: JF825141; CYB: JF825141; ND5: JF825141	[S10]	
Poritidae	*Goniopora djiboutiensis*	Yes	LC		[S10]	
Poritidae	*Goniopora eclipsensis*	Yes	LC		[S10]	
Poritidae	*Goniopora fruticosa*	Yes	LC		[S10]	
Poritidae	*Goniopora lobata*	Yes	NT		[S10]	
Poritidae	*Goniopora minor*	Yes	NT		[S10]	
Poritidae	*Goniopora norfolkensis*	Yes	LC		[S10]	
Poritidae	*Goniopora palmensis*	Yes	LC		[S10]	
Poritidae	*Goniopora pandoraensis*	Yes	LC		[S10]	
Poritidae	*Goniopora pearsoni*	Yes	LC		[S10]	
Poritidae	*Goniopora pendulus*	Yes	LC		[S10]	
Poritidae	*Goniopora planulata*	Yes	VU		[S10]	
Poritidae	*Goniopora polyformis*	Yes	VU		[S10]	
Poritidae	*Goniopora savignyi*	Yes	LC		[S10]	
Poritidae	*Goniopora somaliensis*	Yes	LC		[S10]	
Poritidae	*Goniopora stokesi*	Yes	NT	12S: EF597060; 16S: L76008	[S10]	
Poritidae	*Goniopora stutchburyi*	Yes	LC		[S10]	
Poritidae	*Goniopora sultani*	Yes	LC		[S10]	
Poritidae	*Goniopora tenella*	Yes	NT		[S10]	
Poritidae	*Goniopora tenuidens*	Yes	LC		[S5,S10]	
Poritidae	*Machadoporites tantillus*	Yes	DD			Closest to Goniopora & Porites [S38]
Poritidae	*Porites annae*	Yes	NT	COI: FJ423965	[S10]	

Family	Genus/Species	Reef	Red List	Molecular sources	Morphological sources	Remarks
Poritidae	*Porites aranetai*	Yes	VU		[S10]	
Poritidae	*Porites arnaudi*	Yes	LC		[S10]	
Poritidae	*Porites astreoides*	Yes	LC	12S: EF597055; COI: AB441242; CYB: AB441327	[S5,S10]	
Poritidae	*Porites attenuata*	Yes	VU		[S10]	
Poritidae	*Porites australiensis*	Yes	LC		[S10]	
Poritidae	*Porites baueri*	Yes	DD		[S10]	
Poritidae	*Porites bernardi*	Yes	LC		[S10]	
Poritidae	*Porites branneri*	Yes	NT	12S: EF597059; COI: AY451380	[S10]	
Poritidae	*Porites brighami*	Yes	LC		[S10]	
Poritidae	*Porites cocosensis*	Yes	VU		[S10]	
Poritidae	*Porites colonensis*	Yes	DD	COI: FJ423972	[S10]	
Poritidae	*Porites columnaris*	Yes	LC		[S10]	
Poritidae	*Porites compressa*	Yes	LC	12S: EF597053; 16S: L76020; COI: FJ423970	[S10]	
Poritidae	*Porites cumulatus*	Yes	VU		[S10]	
Poritidae	*Porites cylindrica*	Yes	NT	COI: FJ423968	[S10]	
Poritidae	*Porites decasepta*	Yes	DD		[S10]	
Poritidae	*Porites deformis*	Yes	NT		[S10]	
Poritidae	*Porites densa*	Yes	NT		[S10]	
Poritidae	*Porites desilveri*	Yes	EN		[S10]	
Poritidae	*Porites divaricata*	Yes	LC	12S: EF597058; COI: FJ423969	[S10]	
Poritidae	*Porites duerdeni*	Yes	LC	COI: FJ423976	[S10]	
Poritidae	*Porites echinulata*	Yes	NT		[S10]	
Poritidae	*Porites ericacea*	Yes	DD		[S10]	
Poritidae	*Porites eridani*	Yes	EN		[S10]	
Poritidae	*Porites evermanni*	Yes	DD	COI: FJ423984	[S10]	
Poritidae	*Porites excavata*	Yes	DD		[S10]	
Poritidae	*Porites flavus*	Yes	DD		[S10]	

Family	Genus/Species	Reef	Red List	Molecular sources	Morphological sources	Remarks
Poritidae	*Porites furcata*	Yes	LC	COI: FJ423988	[S10]	
Poritidae	*Porites harrisoni*	Yes	NT		[S10]	
Poritidae	*Porites heronensis*	Yes	LC		[S10]	
Poritidae	*Porites horizontalata*	Yes	VU		[S10]	
Poritidae	*Porites latistella*	Yes	LC		[S10]	
Poritidae	*Porites lichen*	Yes	LC	COI: FJ423963	[S10]	
Poritidae	*Porites lobata*	Yes	NT	16S: AF550372; COI: FJ423973	[S10]	
Poritidae	*Porites lutea*	Yes	LC	COI: AB441243; CYB: AB441328	[S10]	
Poritidae	*Porites mayeri*	Yes	LC		[S10]	
Poritidae	*Porites monticulosa*	Yes	LC		[S10]	
Poritidae	*Porites murrayensis*	Yes	NT		[S10]	
Poritidae	*Porites myrmidonensis*	Yes	LC		[S10]	
Poritidae	*Porites napopora*	Yes	VU		[S10]	
Poritidae	*Porites negrosensis*	Yes	NT		[S10]	
Poritidae	*Porites nigrescens*	Yes	VU		[S10]	
Poritidae	*Porites nodifera*	Yes	LC		[S10]	
Poritidae	*Porites okinawensis*	Yes	VU	12S: JF825142; 16S: JF825142; AT6: JF825142; COI: JF825142; CYB: JF825142; ND5: JF825142	[S10]	
Poritidae	*Porites ornata*	Yes	EN		[S10]	
Poritidae	*Porites panamensis*	Yes	LC	COI: FJ423990	[S10]	
Poritidae	*Porites porites*	Yes	LC	12S: DQ643837; 16S: DQ643837; AT6: DQ643837; COI: DQ643837; CYB: DQ643837; ND5: DQ643837	[S10]	
Poritidae	*Porites profundus*	Yes	LC		[S10]	
Poritidae	*Porites pukoensis*	Yes	CR		[S10]	

Family	Genus/Species	Reef	Red List	Molecular sources	Morphological sources	Remarks
Poritidae	*Porites randalli*	Yes	DD	COI: FJ423966	[S10]	New species [S39]
Poritidae	*Porites rugosa*	Yes	VU		[S10]	
Poritidae	*Porites rus*	Yes	LC	COI: FJ423979	[S10]	
Poritidae	*Porites sillimaniana*	Yes	VU		[S10]	
Poritidae	*Porites solida*	Yes	LC	COI: FJ423962	[S10]	
Poritidae	*Porites somaliensis*	Yes	NT		[S10]	
Poritidae	*Porites stephensoni*	Yes	NT		[S10]	
Poritidae	*Porites studeri*	Yes	LC		[S10]	
Poritidae	*Porites sverdrupi*	Yes	VU		[S10]	
Poritidae	*Porites tuberculosa*	Yes	VU		[S10]	
Poritidae	*Porites vaughani*	Yes	LC		[S10]	
Poritidae	*Poritipora paliformis*	Yes	VU			Poritidae monophyly assumed (excluding Alveopora)
Poritidae	*Stylaraea punctata*	Yes	DD			Closest to Porites [S6]
Rhizangiidae	*Astrangia poculata*	Yes	LC	12S: DQ643832;<br>AT6: DQ643832;<br>COI: DQ643832;<br>CYB: DQ643832		Astrangia sp. in GenBank
Rhizangiidae	*Astrangia rathbuni*	No	N/A		[S5]	
Siderastreidae	*Anomastraea irregularis*	Yes	VU	COI: AM494870	[S32]	
Siderastreidae	*Coscinaraea columna*	Yes	LC	COI: AB441210;<br>CYB: AB441295	[S32]	
Siderastreidae	*Coscinaraea crassa*	Yes	NT		[S32]	
Siderastreidae	*Coscinaraea exesa*	Yes	LC		[S32]	
Siderastreidae	*Coscinaraea hahazimaensis*	Yes	VU		[S32]	
Siderastreidae	*Coscinaraea marshae*	Yes	LC		[S32]	
Siderastreidae	*Coscinaraea mcneilli*	Yes	LC		[S32]	
Siderastreidae	*Coscinaraea monile*	Yes	LC		[S32]	
Siderastreidae	*Coscinaraea wellsi*	Yes	LC	COI: AM494861		Fungiidae clade [S40]
Siderastreidae	*Craterestrea levis*	Yes	LC			Closest to Coscinaraea [S30]
Siderastreidae	*Horastrea indica*	Yes	VU	COI: AM494864	[S32]	
Siderastreidae	*Psammocora albopicta*	Yes	DD	COI: FM865871	[S32]	

Family	Genus/Species	Reef	Red List	Molecular sources	Morphological sources	Remarks
Siderastreidae	*Psammocora contigua*	Yes	NT	16S: AF550371; COI: AB441209; CYB: AB441294	[S32]	
Siderastreidae	*Psammocora decussata*	Yes	DD		[S32]	Psammocora monophyly shown [S41]
Siderastreidae	*Psammocora digitata*	Yes	NT	COI: AM494855	[S32]	
Siderastreidae	*Psammocora explanulata*	Yes	LC	COI: AM494845		Fungiidae clade [S40]
Siderastreidae	*Psammocora haimeana*	Yes	LC	COI: FM865874	[S32]	
Siderastreidae	*Psammocora interstinctus*	Yes	DD		[S32]	Psammocora monophyly shown [S41]
Siderastreidae	*Psammocora nierstraszi*	Yes	LC	COI: AM494851	[S32]	
Siderastreidae	*Psammocora obtusangula*	Yes	NT		[S32]	
Siderastreidae	*Psammocora profundacella*	Yes	LC	COI: AM494853	[S32]	
Siderastreidae	*Psammocora ramosa*	Yes	DD		[S32]	Psammocora monophyly shown [S41]
Siderastreidae	*Psammocora stellata*	Yes	VU		[S32]	Psammocora monophyly shown [S41]
Siderastreidae	*Psammocora superficialis*	Yes	LC		[S32]	
Siderastreidae	*Psammocora vaughani*	Yes	NT		[S32]	
Siderastreidae	*Psammocora verrilli*	Yes	DD		[S32]	Psammocora monophyly shown [S41]
Siderastreidae	*Pseudosiderastrea tayami*	Yes	NT	COI: AM494866	[S32]	
Siderastreidae	*Siderastrea glynni*	Yes	CR		[S32]	Siderastrea monophyly shown [S23]
Siderastreidae	*Siderastrea radians*	Yes	LC	12S: DQ643838; 16S: DQ643838; AT6: DQ643838; COI: DQ643838; CYB: DQ643838; ND5: DQ643838	[S32]	
Siderastreidae	*Siderastrea savignyana*	Yes	LC	COI: AB441215; CYB: AB441300	[S32]	
Siderastreidae	*Siderastrea siderea*	Yes	LC	12S: EF597067; COI: AB441211; CYB: AB441296	[S32]	
Siderastreidae	*Siderastrea stellata*	Yes	DD	COI: AB441213; CYB: AB441298	[S5,S32]	
Stenocyathidae	*Stenocyathus vermiformis*	No	N/A	COI: HM018619		
Trachyphylliidae	*Trachyphyllia geoffroyi*	Yes	NT	COI: AB117287; CYB: AB117372		Clade VII-B monophyly shown [S18]

Family	Genus/Species	Reef	Red List	Molecular sources	Morphological sources	Remarks
Turbinoliidae	*Alatotrochus rubescens*	No	N/A		[S42]	
Turbinoliidae	*Australocyathus vincentinus*	No	N/A		[S42]	
Turbinoliidae	*Conocyathus*	No	N/A		[S42]	
Turbinoliidae	*Cryptotrochus*	No	N/A		[S42]	
Turbinoliidae	*Cyathotrochus pileus*	No	N/A	12S: EF597069; COI: HM018623	[S42]	
Turbinoliidae	*Deltocyathoides*	No	N/A		[S42]	
Turbinoliidae	*Dunocyathus*	No	N/A		[S42]	
Turbinoliidae	*Endocyathopora laticostata*	No	N/A		[S42]	
Turbinoliidae	*Foveolocyathus*	No	N/A		[S42]	
Turbinoliidae	*Holcotrochus*	No	N/A		[S42]	
Turbinoliidae	*Idiotrochus*	No	N/A		[S42]	
Turbinoliidae	*Kionotrochus suteri*	No	N/A		[S42]	
Turbinoliidae	*Notocyathus*	No	N/A	12S: EF597061; 16S: AF265584	[S42]	Notocyathus sp. in GenBank
Turbinoliidae	*Peponocyathus*	No	N/A		[S42]	
Turbinoliidae	*Platytrochus*	No	N/A		[S42]	
Turbinoliidae	*Pleotrochus*	No	N/A		[S42]	
Turbinoliidae	*Pseudocyathoceras avis*	No	DD		[S42]	
Turbinoliidae	*Sphenotrochus*	No	N/A		[S42]	
Turbinoliidae	*Thrypticotrochus petterdi*	No	N/A		[S42]	
Turbinoliidae	*Trematotrochus*	No	N/A		[S42]	
Turbinoliidae	*Tropidocyathus labidus*	No	N/A	12S: EF597062; 16S: AF265585	[S42]	
Turbinoliidae	*Tropidocyathus lessoni*	No	N/A	COI: HM018669	[S42]	
Turbinoliidae	*Turbinolia stephensoni*	No	N/A		[S42]	

Table S1 References

S1 Wallace, C. C. 1999 *Staghorn Corals of the World: A Revision of the Coral Genus* Acropora. Collingwood: CSIRO Publishing.

S2 Wallace, C. C., Turak, E. & DeVantier, L. M. 2011 Novel characters in a conservative coral genus: three new species of *Astreopora* (Scleractinia: Acroporidae) from West Papua. *J. Nat. Hist.* **45**, 1905–1924. (doi:10.1080/00222933.2011.573098)

S3 Ditlev, H. 2003 New scleractinian corals (Cnidaria: Anthozoa) from Sabah, North Borneo. Description of one new genus and eight new species, with notes on their taxonomy and ecology. *Zool. Meded. Leiden* **77**, 193–219.

S4 Wallace, C. C., Chen, C. A., Fukami, H. & Muir, P. R. 2007 Recognition of separate genera within *Acropora* based on new morphological, reproductive and genetic evidence from *Acropora togianensis*, and elevation of the subgenus *Isopora* Studer, 1878 to genus (Scleractinia: Astrocoeniidae; Acroporidae). *Coral Reefs* **26**, 231–239. (doi:10.1007/s00338-007-0203-4)

S5 Pires, D. O. & Castro, C. B. 1997 Scleractinia and Corallimorpharia: An analysis of cnidae affinity. *Proc. 8th Int. Coral Reef Symp.* **2**, 1581–1586.

S6 Veron, J. E. N. 1986 *Corals of Australia and the Indo-Pacific*. Sydney: Angus & Robertson.

S7 Veron, J. E. N. 1990 New Scleractinia from Japan and other Indo-West Pacific countries. *Galaxea* **9**, 95–173.

S8 Veron, J. E. N. 2000 *Corals of the World*. Townsville: Australian Institute of Marine Science.

S9 Veron, J. E. N. 2002 *New Species Described in Corals of the World*. Townsville: Australian Institute of Marine Science.

S10 Daly, M., Fautin, D. G. & Cappola, V. A. 2003 Systematics of the Hexacorallia (Cnidaria: Anthozoa). *Zool. J. Linn. Soc.* **139**, 419–437. (doi:10.1046/j.1096-3642.2003.00084.x)

S11 Locke, J. M., Weil, E. & Coates, K. A. 2007 A newly documented species of *Madracis* (Scleractinia: Pocilloporidae) from the Caribbean. *Proc. Biol. Soc. Wash.* **120**, 214–226. (doi:10.2988/0006-324X(2007)120[214:ANDSOM]2.0.CO;2)

S12 Yabe, H. & Sugiyama, T. 1941 Recent reef-building corals from Japan and the south sea islands under the Japanese mandate. II. *Sci. Rep. Tôhoku Imp. Univ. 2nd Ser. (Geol.) Spec. Vol.* **2**, 67–91.

S13 Cairns, S. D. 2001 A generic revision and phylogenetic analysis of the Dendrophylliidae (Cnidaria: Scleractinia). *Smithsonian Contrib. Zool.* **615**, 1–75.

S14 Carpenter, K. E. et al. 2008 One-third of reef-building corals face elevated extinction risk from climate change and local impacts. *Science* **321**, 560–563. (doi:10.1126/science.1159196)

S15 Chevalier, J.-P. 1971 Les scléractiniaires de la Mélanésie Francaise (Nouvelle Calédonie, Iles Chesterfield, Iles Loyauté, Nouvelles Hébrides). Première partie. *Expéd. Française Récifs Coralliens Nouvelle Calédonie* **5**, 1–307.

S16 Lin, M.-F., Luzon, K. S., Licuanan, W. Y., Ablan-Lagman, M. C. & Chen, C. A. 2011 Seventy-four universal primers for characterizing the complete mitochondrial genomes of scleractinian corals (Cnidaria; Anthozoa). *Zool. Stud.* **50**, 513–524.

S17 Veron, J. E. N., Pichon, M. & Wijsman-Best, M. 1977 *Scleractinia of Eastern Australia. Part II. Families Faviidae, Trachyphylliidae.* Townsville: Australian Institute of Marine Science.

S18 Huang, D., Licuanan, W. Y., Baird, A. H. & Fukami, H. 2011 Cleaning up the 'Bigmessidae': molecular phylogeny of scleractinian corals from Faviidae, Merulinidae, Pectiniidae and Trachyphylliidae. *BMC Evol. Biol.* **11**, 37. (doi:10.1186/1471-2148-11-37)

S19 Wijsman-Best, M. 1972 Systematics and ecology of New Caledonian Faviinae (Coelenterata – Scleractinia). *Contrib. Zool.* **42**, 3–90.

S20 Budd, A. F. & Smith, N. D. 2005 Diversification of a new Atlantic clade of scleractinian reef corals: insights from phylogenetic analysis of morphologic and molecular data. *Paleontol. Soc. Pap.* **11**, 103–128.

S21 Moll, H. & Best, M. B. 1984 New scleractinian corals (Anthozoa: Scleractinia) from the Spermonde Archipelago, South Sulawesi, Indonesia. *Zool. Meded. Leiden* **58**, 47–58.

S22 Scheer, G. & Pillai, C. S. G. 1983 Report on the stony corals from the Red Sea. *Zoologica* **131**, 1–198.

S23 Kitahara, M. V., Cairns, S. D., Stolarski, J., Blair, D. & Miller, D. J. 2010 A comprehensive phylogenetic analysis of the Scleractinia (Cnidaria, Anthozoa) based on mitochondrial CO1 sequence data. *PLoS ONE* **5**, e11490. (doi:10.1371/journal.pone.0011490)

S24 Hoeksema, B. W. 1989 Taxonomy, phylogeny and biogeography of mushroom corals (Scleractinia: Fungiidae). *Zool. Verh. Leiden* **254**, 1–295.

S25 Gittenberger, A., Reijnen, B. T. & Hoeksema, B. W. 2011 A molecularly based phylogeny reconstruction of mushroom corals (Scleractinia: Fungiidae) with taxonomic consequences and evolutionary implications for life history traits. *Contrib. Zool.* **80**, 107–132.

S26 Hoeksema, B. W. 1993 Historical biogeography of *Fungia* (*Pleuractis*) spp. (Scleractinia: Fungiidae), including a new species from the Seychelles. *Zool. Meded. Leiden* **67**, 639–654.

S27 Hoeksema, B. W. 2009 Attached mushroom corals (Scleractinia: Fungiidae) in sediment-stressed reef conditions at Singapore, including a new species and a new record. *Raffles Bull. Zool.* **S22**, 81–90.

S28 Vaughan, T. W. 1901 The stony corals of the Porto Rican waters. *Bull. U.S. Fish Commiss. 1900* **2**, 289–320.

S29 Pinzón, J. H. & Weil, E. 2011 Cryptic species within the Atlantic-Caribbean genus *Meandrina* (Scleractinia): a multidisciplinary approach and description of the new species *Meandrina jacksoni*. *Bull. Mar. Sci.* **87**, 823–853. (doi:10.5343/bms.2010.1085)

S30 Head, S. M. 1983 An undescribed species of *Merulina* and a new genus and species of siderastreid coral from the Red Sea. *J. Nat. Hist.* **17**, 419–435. (doi:10.1080/00222938300770281)

S31 Veron, J. E. N. & Pichon, M. 1980 *Scleractinia of Eastern Australia. Part III. Families Agariciidae, Siderastreidae, Fungiidae, Oculinidae, Merulinidae, Mussidae, Pectiniidae, Caryophylliidae, Dendrophylliidae.* Townsville: Australian Institute of Marine Science.

S32 Pandolfi, J. M. 1992 Successive isolation rather than evolutionary centres for the origination of Indo-Pacific reef corals. *J. Biogeogr.* **19**, 593–609. (doi:10.2307/2845703)

S33 Best, M. B. & Hoeksema, B. W. 1987 New observations on scleractinian corals from Indonesia: 1. Free-living species belonging to the Faviina. *Zool. Meded. Leiden* **61**, 387–403.

S34 Fenner, D. P. 1993 Species distinctions among several Caribbean stony corals. *Bull. Mar. Sci.* **53**, 1099–1116.

S35 Claereboudt, M. R. 1990 *Galaxea paucisepta* nom. nov. (for *G. pauciradiata*), rediscovery and redescription of a poorly known scleractinian species (Oculinidae). *Galaxea* **9**, 1–8.

S36 Wells, J. W. 1937 New genera of Mesozoic and Cenozoic corals. *J. Paleontol.* **11**, 73–77.

S37 Fukami, H. et al. 2008 Mitochondrial and nuclear genes suggest that stony corals are monophyletic but most families of stony corals are not (Order Scleractinia, Class Anthozoa, Phylum Cnidaria). *PLoS ONE* **3**, e3222. (doi:10.1371/journal.pone.0003222)

S38 Claereboudt, M. R. & Al-Amri, I. S. 2004 *Calathiscus tantillus*, a new genus and new species of scleractinian coral (Scleractinia, Poritidae) from the Gulf of Oman. *Zootaxa* **532**, 1–8.

S39 Forsman, Z. H. & Birkeland, C. 2009 *Porites randalli*: a new coral species (Scleractinia, Poritidae) from American Samoa. *Zootaxa* **2244**, 51–59.

S40 Benzoni, F., Stefani, F., Stolarski, J., Pichon, M., Mitta, G. & Galli, P. 2007 Debating phylogenetic relationships of the scleractinian *Psammocora*: molecular and morphological evidences. *Contrib. Zool.* **76**, 35–54.

S41 Benzoni, F., Stefani, F., Pichon, M. & Galli, P. 2010 The name game: morpho-molecular species boundaries in the genus *Psammocora* (Cnidaria, Scleractinia). *Zool. J. Linn. Soc.* **160**, 421–456. (doi:10.1111/j.1096-3642.2010.00622.x)

S42 Cairns, S. D. 1997 A generic revision and phylogenetic analysis of the Turbinoliidae (Cnidaria: Scleractinia). *Smithsonian Contrib. Zool.* **591**, 1–55.
